# The quail genome: insights into social behaviour, seasonal biology and infectious disease response

**DOI:** 10.1186/s12915-020-0743-4

**Published:** 2020-02-12

**Authors:** Katrina M. Morris, Matthew M. Hindle, Simon Boitard, David W. Burt, Angela F. Danner, Lel Eory, Heather L. Forrest, David Gourichon, Jerome Gros, LaDeana W. Hillier, Thierry Jaffredo, Hanane Khoury, Rusty Lansford, Christine Leterrier, Andrew Loudon, Andrew S. Mason, Simone L. Meddle, Francis Minvielle, Patrick Minx, Frédérique Pitel, J. Patrick Seiler, Tsuyoshi Shimmura, Chad Tomlinson, Alain Vignal, Robert G. Webster, Takashi Yoshimura, Wesley C. Warren, Jacqueline Smith

**Affiliations:** 1 The Roslin Institute and R(D)SVS, University of Edinburgh, Easter Bush, Midlothian, EH25 9RG UK; 2 GenPhySE, Université de Toulouse, INRAE, ENVT, 31326 Castanet Tolosan, France; 3 The John Hay Building, Queensland Biosciences Precinct, 306 Carmody Road, The University of Queensland, QLD, St Lucia, 4072 Australia; 4 Virology Division, Department of Infectious Diseases, St. Jude Children’s Research Hospital, 262 Danny Thomas Place, Memphis, TN 38105 USA; 5 PEAT Pôle d’Expérimentation Avicole de Tours, Centre de recherche Val de Loire, INRAE, 1295 Nouzilly, UE France; 6 Department of Developmental and Stem Cell Biology, Institut Pasteur, 25 rue du Docteur Roux, 75724, Cedex 15 Paris, France; 7 CNRS URA3738, 25 rue du Dr Roux, 75015 Paris, France; 8 McDonnell Genome Institute, Washington University School of Medicine, 4444 Forest Park Blvd, St Louis, MO 63108 USA; 9 CNRS UMR7622, Inserm U 1156, Laboratoire de Biologie du Développement, Sorbonne Université, IBPS, 75005 Paris, France; 10 Department of Radiology and Developmental Neuroscience Program, Saban Research Institute, Children’s Hospital Los Angeles and Keck School of Medicine of the University of Southern California, Los Angeles, CA 90027 USA; 11 UMR85 Physiologie de la Reproduction et des Comportements, INRAE, CNRS, Université François Rabelais, IFCE, INRAE, Val de Loire, 37380 Nouzilly, Centre France; 12 Centre for Biological Timing, Faculty of Biology, Medicine and Health, School of Medical Sciences, University of Manchester, 3.001, A.V. Hill Building, Oxford Road, Manchester, M13 9PT UK; 13 GABI, INRAE, AgroParisTech, Université Paris-Saclay, 78350 Jouy-en-Josas, France; 14 Department of Biological Production, Tokyo University of Agriculture and Technology, 3-8-1 Harumi-cho, Fuchu, Tokyo, 183-8538 Japan; 15 Institute of Transformative Bio-Molecules (WPI-ITbM), Nagoya University, Furo-cho, Chikusa-ku, Nagoya, 464-8601 Japan; 16 Department of Animal Sciences, Department of Surgery, Institute for Data Science and Informatics, University of Missouri, Bond Life Sciences Center, 1201 Rollins Street, Columbia, MO 65211 USA

**Keywords:** *Coturnix japonica*, Quail, Genome, Influenza, Seasonality, Photoperiod, Bird flu, H5N1

## Abstract

**Background:**

The Japanese quail (*Coturnix japonica*) is a popular domestic poultry species and an increasingly significant model species in avian developmental, behavioural and disease research.

**Results:**

We have produced a high-quality quail genome sequence, spanning 0.93 Gb assigned to 33 chromosomes. In terms of contiguity, assembly statistics, gene content and chromosomal organisation, the quail genome shows high similarity to the chicken genome. We demonstrate the utility of this genome through three diverse applications. First, we identify selection signatures and candidate genes associated with social behaviour in the quail genome, an important agricultural and domestication trait. Second, we investigate the effects and interaction of photoperiod and temperature on the transcriptome of the quail medial basal hypothalamus, revealing key mechanisms of photoperiodism. Finally, we investigate the response of quail to H5N1 influenza infection. In quail lung, many critical immune genes and pathways were downregulated after H5N1 infection, and this may be key to the susceptibility of quail to H5N1.

**Conclusions:**

We have produced a high-quality genome of the quail which will facilitate further studies into diverse research questions using the quail as a model avian species.

**Electronic supplementary material:**

The online version of this article (10.1186/s12915-020-0743-4) contains supplementary material, which is available to authorized users.

## Background

Japanese quail (*Coturnix japonica*) is a migratory bird indigenous to East Asia and is a popular domestic poultry species raised for meat and eggs in Asia and Europe. Quail have been used in genetics research since 1940 [1] and are an increasingly important model in developmental biology, behaviour and biomedical studies [2]. Quail belong to the same family as chickens (Phasianidae) but have several advantages over chickens as a research model. They are small and easy to raise, have a rapid growth rate and a short life cycle, becoming sexually mature only 7 to 8 weeks after hatching [3]. Quail are key for comparative biology research among Galliformes, showing key differences to chickens and other model fowl species, including migratory and seasonal behaviour and immune function [2].

Quail have become a key model in several research fields [4]. The avian embryo has long been a popular model for studying developmental biology due to the accessibility of the embryo, which permits fate mapping studies [5, 6] and dynamic imaging of embryogenesis [7–9]. Several transgenic lines that express fluorescent proteins now exist, which greatly facilitates time-lapse imaging and tissue transplantation [7, 10–13].

The quail embryo survives manipulation and culture better than chicken embryos making them ideal for this type of research [3]. Quail have been used as a model for stem cell differentiation, for example a culture system that mimics the development of haematopoietic stem cells has been recently developed, as quail show greater cell multiplication in these cultures than chickens [14].

Quail are also used to study the genetics underlying social behaviours [15], sexual behaviour [16, 17], pre- and post-natal stress programming [18] and emotional reactivity [19–22]. Japanese quail have a fast and reliable reproductive response to increased photoperiod, making them an important model species for investigation into seasonal behaviour and reproduction in birds [23–25]. The molecular mechanisms behind seasonality including metabolism and growth, immunity, reproduction, behaviour and feather moult is poorly understood despite its importance in the management of avian species.

Quail are also important in disease research [26]. Different strains of quail have been developed as models of human disease such as albinism [27] or necrotizing enterocolitis in neonates [28]. Quail lines have also been selected on their immunological response [29]. There are key differences in the immunogenetics of quail and chicken—particularly in the major histocompatibility complex (MHC) [30, 31]. Investigating the immunology of quail is important for understanding infectious disease spread and control in poultry. For example they are an important species for influenza transmission, with previous research showing that quail may play a key role as an intermediate host in evolution of avian influenza [32–34]. Zoonotic H5N1 influenza strains have crossed from quail to human causing mortality in the past [35, 36], making them a potential pandemic source.

We have produced a high-quality annotated genome of the Japanese quail (*Coturnix japonica*) and herein describe the assembly and annotation of the quail genome and demonstrate key uses of the genome in immunogenetics, disease, seasonality and behavioural research demonstrating its utility as an avian model species.

## Results

### Genome assembly and annotation

Using an Illumina HiSeq 2500 instrument, we sequenced a male *Coturnix japonica* individual from a partially inbred quail line (*F* > 0.6), obtained through four generations of full-sib mating from a partially inbred base population. Total sequence genome input coverage of Illumina reads was ~ 73×, using a genome size estimate of 1.1 Gb. Additionally, 20× coverage of long PacBio reads were sequenced and used to close gaps. The male genome *Coturnix japonica* 2.0 was assembled using ALLPATHS2 software [37] and is made up of a total of 2531 scaffolds (including single contigs with no scaffold association) with an N50 scaffold length of 2.9 Mb (N50 contig length is 511 kb). The assembly sequence size is 0.927 Gb with only 1.7% (16 Mb) not assigned to 33 total chromosomes. *Coturnix japonica* 2.0 assembly metrics were comparable to previous assemblies of Galliformes, and superior to other genomes of other quail species [38, 39] in ungapped (contigs) sequence length metrics (Table 1). Specifically, in comparison to recently published genomic data from the Japanese quail [39], our genome is substantially less fragmented (contig N50 of 0.511 Mb vs 0.027 Mb), has been assigned to more chromosomes and has more complete annotation with ncRNA, mRNA and pseudogenes predicted. Our estimate of total interspersed repetitive elements was 19% genome-wide based on masking with Windowmasker [40]. In the genomes of other quail species, the estimated repeat content was much lower, ~ 10% less in both species [38].

**Table 1:** Representative assembly metrics for sequenced Galliform genomes

Common name	Assembled version	N50 contig (Mb)	N50 scaffold (Mb)	Total assembly size (Gb)	Assembled chromosomes
Japanese quail	*Coturnix japonica* 2.0	0.511	3.0	0.93	33
Japanese quail	Wu et al. PMID: 29762663	0.027	1.8	0.90	30
Chicken	*Gallus gallus* 5.0	2.895	6.3	1.20	34
Scaled quail	ASM221830v1	0.154	1.0	1.01	NA
Northern bobwhite	ASM59946v2	0.056	2.0	1.13	NA
Turkey	Turkey 5.0	0.036	3.8	1.13	33

All species-specific assembly metrics derived from the NCBI assembly archive

To improve the quantity and quality of data used for the annotation of the genome, we sequenced RNA extracted from seven tissues sampled from the same animal used for the genome assembly. Using the same inbred animal increases the alignment rate and accuracy. The amount of data produced for annotation from the 7 tissues is (in Gb) as follows: 18.9 in brain, 35.6 in heart, 19.3 in intestine, 27.8 in kidney, 39.0 in liver, 18.8 in lung and 34.0 in muscle. High sequencing depth was aimed for in these tissues, to help detect low expression genes including those that are tissue-specific. In total, we predicted 16,057 protein-coding genes and 39,075 transcripts in the *Coturnix japonica* genome (Table 2). In comparison to other assembled and annotated Galliformes, transcript and protein alignments of known chicken RefSeq proteins to *Coturnix japonica* suggest the gene representation is sufficient for all analyses described herein (Table 3). However, we find ~ 1000 fewer protein-coding genes in the Japanese quail than the northern bobwhite (*Colinus virginianus*) and scaled quail (*Callipepla squamata*) genomes [38]. We attribute this to the use of different gene prediction algorithms, and the slightly lower assembled size of Japanese quail, 927 Mb compared to 1 Gb in other quail genomes [38] (Table 1).

**Table 2:** Representative gene annotation measures for assembled Galliform genomes

Common name	Assembled version	Protein-coding genes	Total ncRNA	mRNAs
Japanese quail	*Coturnix japonica* 2.0	16,057	4108	39,075
Japanese quail	Wu et al. PMID: 29762663	16,210	NA	NA
Chicken	*Gallus gallus* 5.0	19,137	6550	46,334
Turkey	Turkey 5.0	18,511	8552	33,308

All species-specific gene annotation metrics derived from the NCBI RefSeq database

**Table 3:** Estimates of gene and protein representation for sequenced Galliform genomes

		Transcript^1^		Protein^2^	
Common name	Assembled version	Average % identity	Average % coverage	Average % identity	Average % coverage
Japanese quail	*Coturnix japonica* 2.0	93.4	96.2	80.0	85.0
Chicken	*Gallus gallus* 5.0	90.4	84.3	78.0	84.6
Turkey	Turkey 5.0	NA	NA	80.7	80.1

^1^Predicted transcripts per species aligned to Aves known RefSeq transcripts (*n* = 8776)

^2^Predicted proteins per species aligned to Aves known RefSeq (*n* = 7733)

For further annotation, a set of genes unnamed by the automated pipeline were manually annotated. As part of an ongoing project to investigate hemogenic endothelium commitment and HSC production [14], transcriptomes were produced for two cultured cell fractions. Study of these cells is critical for developmental biology and regenerative medicine, and quail are an excellent model for studying these as they produce much more haematopoietic cells than similar chicken cultures. Approximately 8000 genes were expressed in these cells lines which lacked gene names or annotation from the automated annotation pipeline. Using BLAST [41] searches to identify homology to other genes, 3119 of these were manually annotated (Additional file 1).

Genome completeness was also quantitatively assessed by analysing 4915 single-copy, orthologous genes derived from OrthoDB v7 and v9 [42]. Presence and contiguity of these conserved, avian-specific genes were tested with BUSCO v3.0.2 [43]. A comparison with the chicken assembly [44] (*Gallus gallus* 5.0) indicates that 95% of these genes are present and full length in all three assemblies. The percentage of duplicated, fragmented and missing genes are also very similar between the assemblies (Additional file 2: Figure S1). The quail genome has 10 more missing and 23 more fragmented genes than the *Gallus gallus* 5.0 assembly. However, relative to the total number of genes in the benchmarking set, these increases amount to just 0.2% and 0.5%, respectively. This indicates that the quail genome, like the chicken genome, is highly contiguous and, in terms of its expected gene content, is close to complete.

### Galliforme genome synteny

**Fig. 1:**
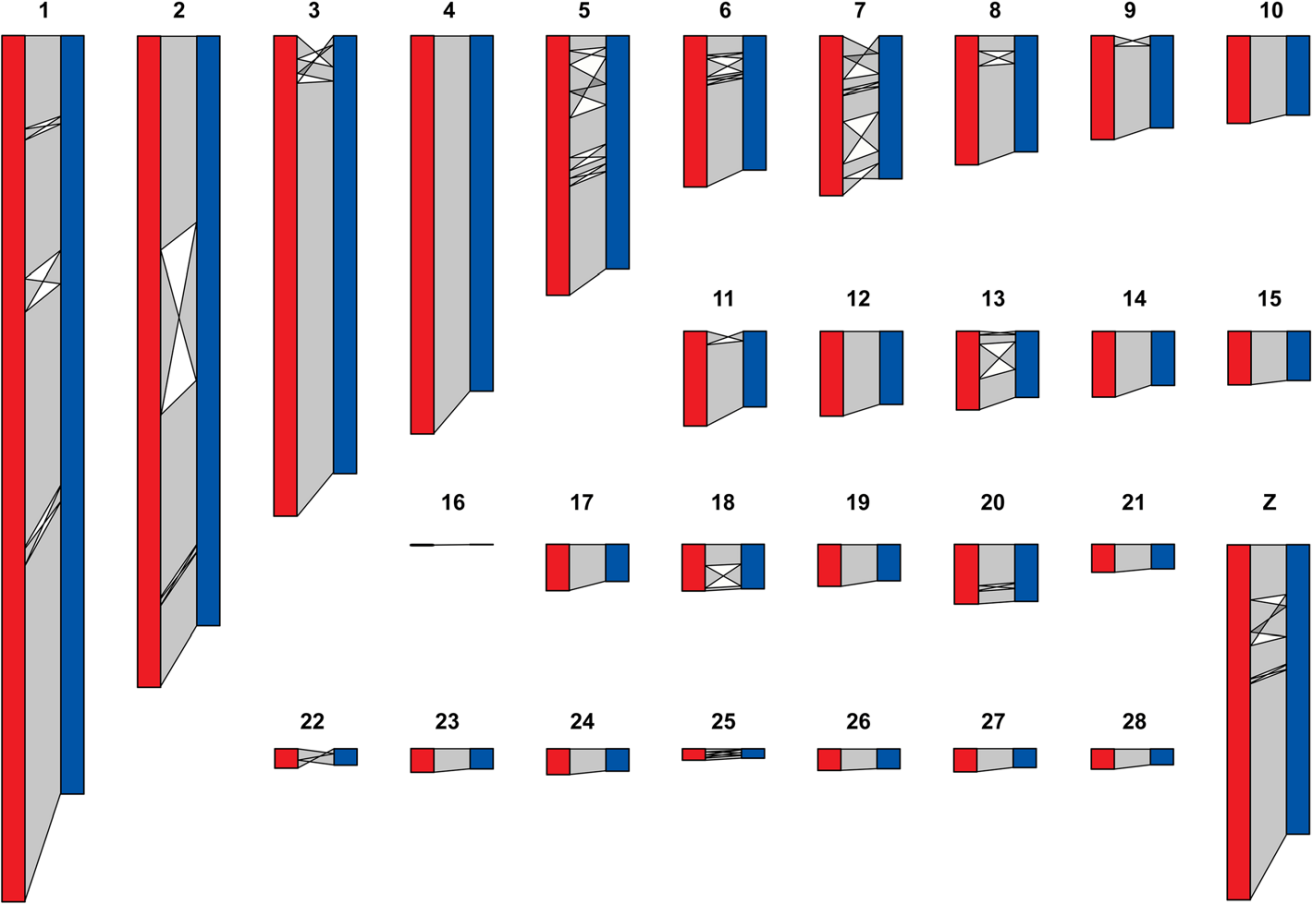
Synteny map of chicken (red) and quail (blue) chromosomes

Comparative mapping of the quail and chicken genomes revealed a high conservation of the chromosomal arrangement (Fig. 1; Additional file 3), with no major rearrangements since the divergence of the two species approximately 23 MYA [45]. All identified quail chromosomes showed synteny conservation to their chicken chromosomal counterparts. By comparison, the turkey (*Meleagris gallopavo*) genome is more highly rearranged with two chromosomes having synteny conservation to each of chicken and quail chromosomes 2 and 4 [46]. No large intra-chromosomal translocations were seen between chicken and quail chromosomes, compared to the two seen in the turkey [46, 47]. Inversions and inter-chromosomal translocations were common, with 33 large (> 1 Mb) inversions or translocations occurring between chicken and quail chromosomes (Fig. 1; Additional file 3). The quail chromosomes are more compact than their chicken and turkey counterparts (14% smaller on average). This may be linked to the metabolic cost of migratory flight in quails, as previous studies have demonstrated smaller genomes and higher deletion rates in flying birds compared to flightless birds [48].

Orthologous genes between quail and closely related species were identified through reciprocal BLAST searches. One-to-one orthologs in chicken were identified for 78.2% of all quail genes and 91.8% of protein-coding quail genes (Additional file 4), indicating a high degree of genic conservation in the quail genome. Fewer orthologs were seen between turkey and quail genes (69.3%), although the number of orthologs of protein-coding genes was similar (91.7%), so the discrepancy is likely due to missing non-coding gene predictions in the turkey genome. As expected, conservation of one-to-one orthologs was lower with the mallard duck (*Anas platyrhynchos*), with duck orthologs identified for 64.5% of quail genes (78.9% protein-coding genes).

### Endogenous retroviruses (ERVs)

ERVs represent retroviral integrations into the germline over millions of years and are the only long terminal repeat (LTR) retrotransposons which remain in avian genomes [49, 50]. While the majority of ERVs have been degraded or epigenetically silenced, more recent integrations retain the ability to produce retroviral proteins, impacting the host immune response to novel exogenous infections [51, 52]. A total of 19.4 Mb of the *Coturnix japonica* 2.0 assembly was identified as ERV sequence using the LocaTR pipeline [49] (Additional file 5 and Additional file 6). ERVs therefore account for 2.1% of the quail genome sequence, levels similar to those in the chicken and turkey [44] (Additional file 7), and similarly analysed passerine birds [49].

The majority of ERV sequences in all three genomes were short and fragmented, but 393 intact ERVs were identified in the quail, most of which were identified as alpha-, beta- or gamma-retroviral sequences by reverse transcriptase homology. It is possible that the smaller genome size of the quail compared to other birds reflects a more limited expansion of ERVs and other repeats (such as the LINE CR1 element; Additional file 7) within the genome, following the basal avian lineage genome contraction [48, 50]. However, ERV content is highly species-specific [49].

Despite variation in total and intact ERV content, the overall genomic ERV distribution in these three gallinaceous birds was highly similar. ERV sequence density was strongly correlated with chromosome length on the macrochromosomes and Z chromosome (*r* > 0.97; *P* < 0.001), but there was no significant correlation across the other smaller chromosomes. Furthermore, ERV density on each Z chromosome was at least 50% greater than would be expected on an autosome of equal length. These results support the depletion of repetitive elements in gene dense areas of the genome, and the persistence of insertions in poorly recombining regions, as was seen in the chicken [49]. This is further supported by the presence of clusters of intact ERVs (where density was five times the genome-wide level) on the macrochromosomes and sex chromosomes (Additional file 7).

### Selection for social motivation

Quail has been used as a model to study the genetic determinism of behaviour traits such as social behaviours and emotional reactivity [21, 22, 53], these being major factors in animal adaptation. Moreover, quail selected with a low social motivation behave in a way that can be related to autistic-like traits, so the genes and causal variants are of wider interest to the biomedical community. Here we use the new quail genome assembly to improve previous results on the detection of selection signatures in lines selected for sociability. Due to the non-availability of a useable quail reference genome at the start of these studies, genomic sequence data produced from two DNA pools of 10 individuals each from two quail lines diverging for social motivation had been aligned to the chicken reference genome, GallusWU2.58 [54]. As a result, only 55% of the reads had mapped in proper pairs, whereas by using our quail genome as a reference, this number increased to 92%. This corresponds to an improvement of the averaged coverage from 9× to 20× and of the number of analysed SNPs from 12,364,867 to 13,506,139.

The FLK [55] and local [54] score analysis led to the detection of 32 significant selection signature regions (*p* < 0.05) (Additional file 8); Additional file 2: Figure S2 shows an example of such a region on Chr20. This represents a substantial improvement in the number of detected regions, compared with the 10 regions obtained when using the chicken genome as a reference [54]. Of the 32 detected regions, six may be merged in pairs due to their physical proximity, four regions map to new linkage groups absent in the previous analysis, and eight correspond with results obtained in the previous study (Additional file 8). Altogether, 17 new regions were detected. Of these, eight could be seen in the previous analysis, but had not been considered as they did not reach the significance threshold, and nine are solely due to the availability of our quail assembly. Two very short selection signatures previously detected using the chicken assembly as reference are not recovered here and were most probably false positives.

These results confirm the selection signature regions harbouring genes involved in human autistic disorders or being related to social behaviour [54] (*PTPRE*, *ARL13B*, *IMPK*, *CTNNA2*). Among the genes localised in the newly detected genomic regions, several have also been shown to be implicated in autism spectrum disorders or synaptogenic activity (Additional file 8): mutations in the *EEF1A2* gene (eukaryotic elongation factor 1, alpha-2) have been discovered in patients with autistic behaviours [56]; *EHMT1* (Euchromatin Histone Methyltransferase 1) is involved in autistic syndrome and social behaviour disorders in human and mouse [56–59]; *LRRTM4* (Leucine Rich Repeat Transmembrane Neuronal 4) is a synapse organising protein, member of the *LRRTM* family, involved in mechanisms underlying experience-dependent synaptic plasticity [60].

### A model for avian seasonal biology

Quail is an important model for studying seasonal biology. Seminal work in quail established that pineal melatonin [61, 62] is regulated by the circadian clock [63]. In mammals, photo-sensing is dependent on a single retinal photoreceptor melanopsin (*OPN4*) that regulates pineal melatonin release. Nocturnal melatonin is critical for mammalian neuroendocrine response to photoperiod and is likely to target melatonin receptors in the *pars tuberalis* [64] (PT). Birds have a distinct non-retinal mechanism for photoreception through deep-brain photoreceptors [65] and melatonin does not appear to be critical for most avian seasonal cycles [66]. The medial basal hypothalamus (MBH) seems to be a critical region for avian perception of photoperiod [67]. There are currently three main candidates for avian deep-brain photoreceptors that communicate the photoperiod signal to seasonal cycles: *OPN4* [68], neuropsin [69] (*OPN5*) and vertebrate ancient [70] (*VA*).

**Fig. 2:**
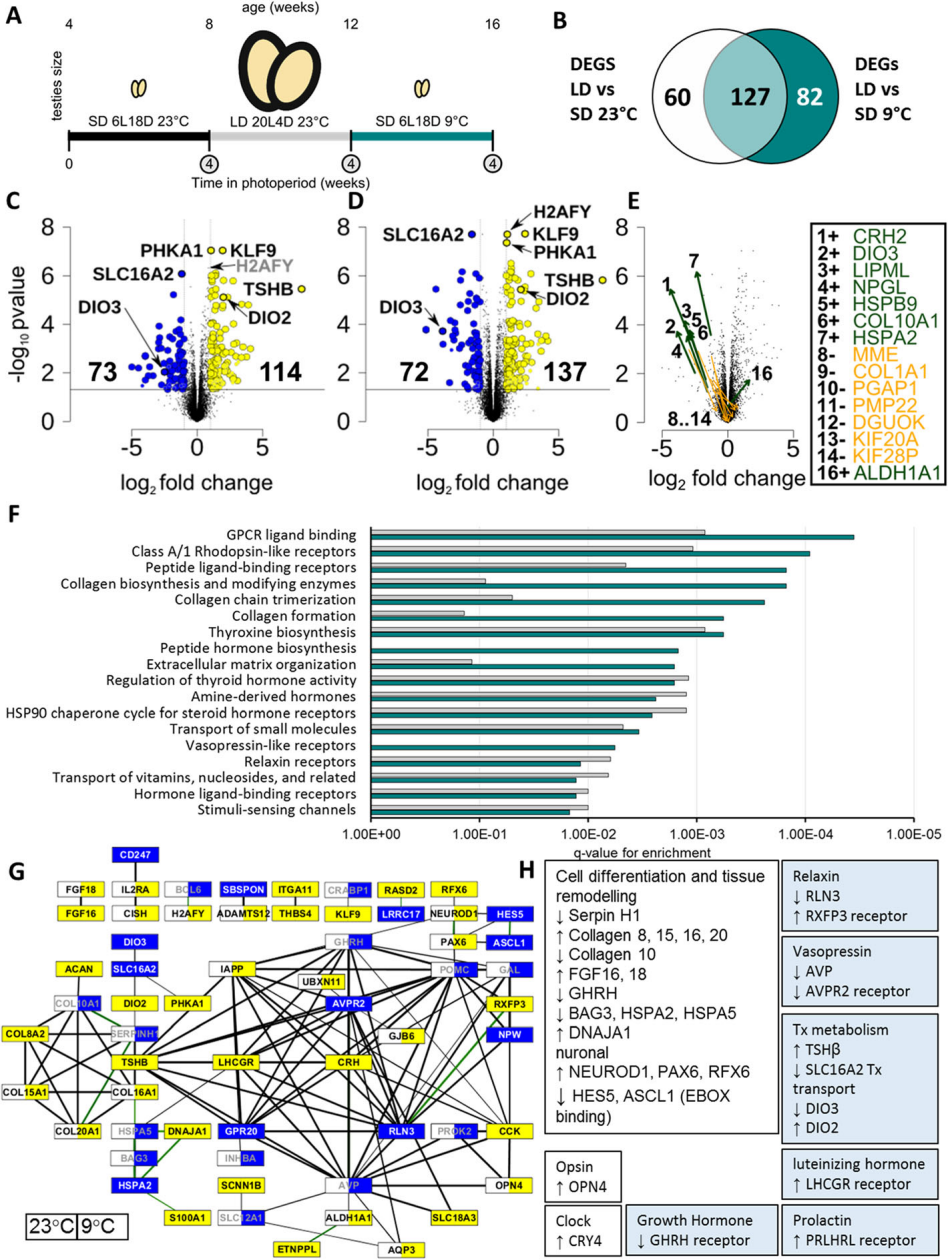
Genome-wide analysis of temperature-dependent transcriptome responses to photoperiod in quail. Experimental design showing the 3 time-points each sampled after 4 weeks of the target photoperiod (circled) with RNA-Seq at *n* = 4 **a**. Intersection of DEGs between LD 23 °C vs SD 23 °C and LD 23 °C vs SD 9 °C **b**. Volcano plots comparing LD 23 °C vs SD 23 °C showing 71 up (yellow) and 42 down (blue) DEGs **c** and LD 23 °C vs SD 23 °C **d**. Grey labels do not pass fold change threshold at 23 °C. Temperature-dependent effects on fold change in DEGs when comparing SD at 23 °C and SD 9 °C. Arrows point from 23 to 9 °C and indicate a significant amplifying (green) or dampening (orange) effect of 9 °C on photoperiod response **e** significantly enriched pathways in DEG genes at LD vs SD 23 °C (grey) and LD vs SD 9 °C (teal) *q*-value thresholds **f**. Network of up (yellow), down (blue) and no significant change (white) regulated inter-connected genes (LD vs SD) using the String database. The left side of a node indicates the expression change at 23 °C and right at 9 °C. Edges are weighted by the combined score, and green edges represent experimental support **g**. Summary of upregulated and downregulated pathways **h**

While melatonin may not be a critical component to avian photoperiod signal transduction, it may play a role. Photoperiodic regulation of gonadotropin-inhibitory hormone (GnIH), first identified in quail, has been shown to be regulated by melatonin [71]. Melatonin receptors are also located in the quail PT [72], and like the mammalian PT [73], the expression of core clock genes in the quail PT [74] are phase-shifted with photoperiod. Previously, two studies [67, 75] have examined temperature-dependent effects of photoperiod on core clock genes, *TSHβ* in the PT and *DIO2* and *DIO3* in the MBH. Here, we leverage the new quail genome for genome-wide analysis to determine how photoperiod and temperature interact to determine the MBH transcriptome (Fig. 2a).

We examined the effect of short- (SD) and long-day (LD) photoperiod (SD, 6L18D & LD, 20L4D) and temperature (9 °C and 23 °C) at 12 h after light on (ZT18) (Fig. 2a; Additional file 2: Figure S3) on genome-wide transcription and identified 269 significantly differentially expressed genes (DEGs; FDR < 0.05, log2FC > 1; Additional file 9). A total of 127 DEGs were regulated irrespective of temperature, 60 and 82 DEGs were specific to the contrast with SD 9 °C and 23 °C, respectively. As a single time point was sampled at ZT18, the differential expression reported inevitably captures both circadian effects, such as shifts in phase/period/amplitude, and photoperiod-dependent effects. Resolving photoperiod responses and circadian effects would require a longer time-series with samples across 24 h. Additionally, photoperiod-dependent effects include both acute and expression dependent on the photoperiod history. The ZT18 time point in LD is 12 h after dark and 2 h before dark in SD, so may include acute light-dark photo-perception.

We identified 16 temperature-dependent DEGs with a large modulating effect of temperature (log2FC > 1) (Fig. 2e). With the exception of aldehyde dehydrogenase (*ALDH1A1*), the temperature-dependent photoperiod effected DEGs were downregulated in LD. There was an equal division of genes between temperature-dependent amplification and suppression of LD downregulated genes.

The MBH shows strong TSHβ induction in LD (Fig. 2c, d, log2FC = 7.96 at 9 °C, 8.36 at 23 °C), indicating the stamp contains the adjacent PT as well as the MBH. Previous in situ data [75] support the localisation of TSHβ in the quail PT. Consistent with previous MBH findings [75], we observed significant upregulation of *DIO2* and downregulation of *DIO3*, in LD. We also observed a significant effect of cold (9 °C) in short days as an amplifier of *DIO3* LP downregulation (Fig. 2e, log2FC = − 3.86 at 9 °C, − 2.51 at 23 °C). We were unable to confirm any significant effect of cold on *DIO2*. We note significant photoperiod-dependent downregulation of the thyroid hormone-specific transporter *SLC16A2* in LP that was amplified at 9 °C (log2FC = − 1.19 at 9 °C, − 1.63 at 23 °C).

Differential regulation of G-protein coupled receptor (GPCR) signalling was the most enriched pathway regulated by photoperiod (Fig. 2f; Additional file 10). It also emerged as the largest connecting component within the String interaction network of DEG genes (Fig. 2g). TSHβ itself binds to the GPCR THR [76]. G-protein signalling is also critical for opsin signalling [77]. We also observed transcriptional regulation in other GPCR hormone receptors, including Relaxin, Vasopressin, LH, Prolactin and GH. GnRH is associated with VA opsins in AVT neurones and has been suggested as a photoperiod sensor [70]. We also noted downregulation of the neuronally important GPCR GPR20 (Fig. 2g). In mice, deficiency of GPR20 is associated with hyperactivity and may play a role in cAMP-dependent mitogenesis [78]. There was a strong enrichment of collagen biosynthetic processes and extracellular matrix organisation processes (Fig. 2f) and a large body of genes associated with cell differentiation and development (Fig. 2h).

We observed photoperiod-dependent regulation of a single clock gene, *CRY4*. *CRY4* is upregulated in LP (log2FC = 0.85 at 23 °C, 1.37 at 9 °C). This is consistent with the finding of Yasuo et al. [67] that the expression of *PER2-3*, *CLOCK*, *BMAL1*, *CRY1-2* and *E4BP4* remain stable across photoperiods. CRY4 has recently been the subject of considerable research in migratory birds [79, 80] and the observed variation across photoperiods in a non-migratory Galliform suggest quail could be an interesting model to further investigate SP-dependent non-migratory CRY4 function in the MBH.

**Fig. 3:**
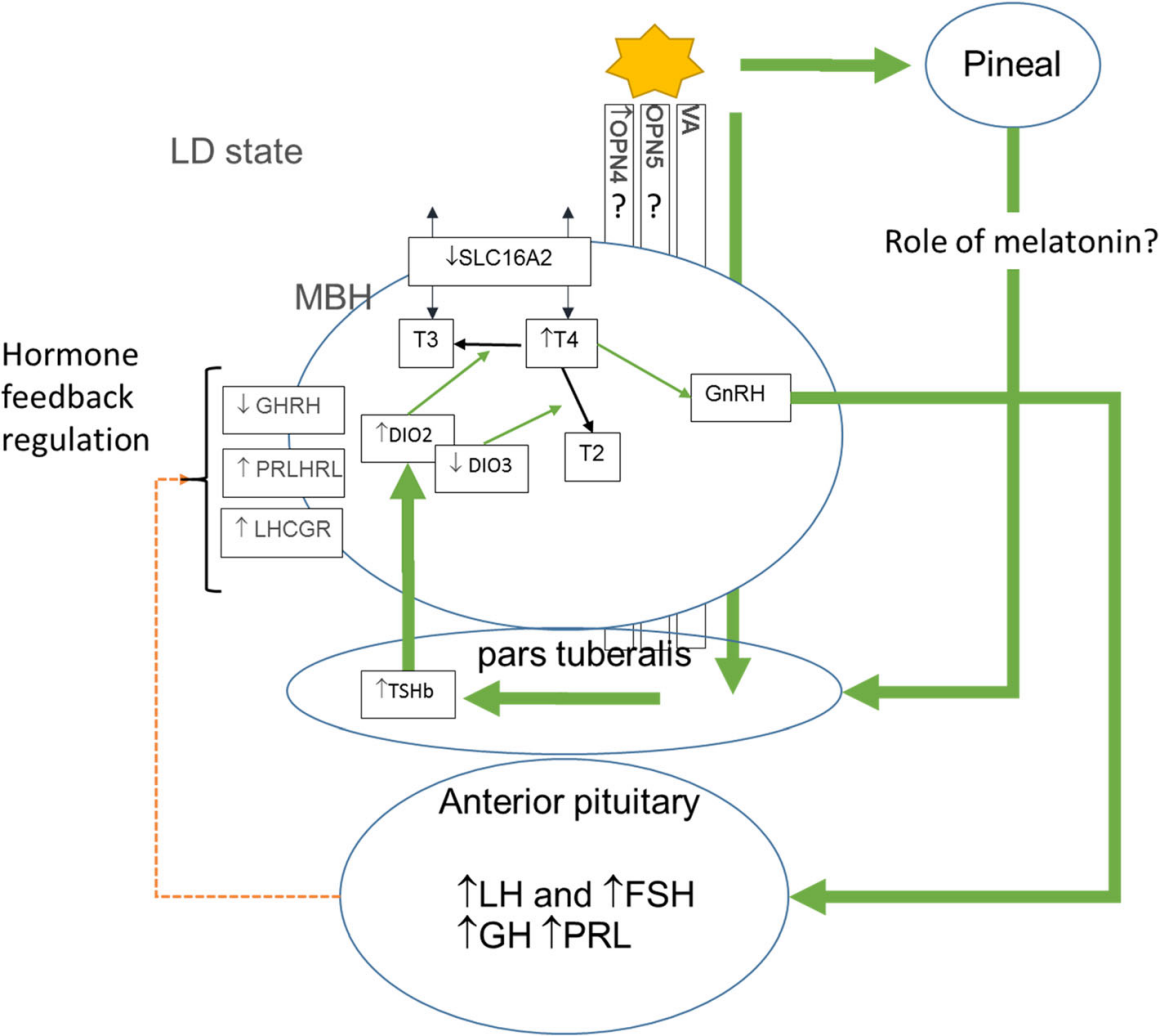
Photoperiod signalling in the MBH incorporating observations from RNA-Seq

We detected photoperiod effects on *OPN4* transcripts, which were upregulated in LD. Photoperiod-dependent expression in *OPN4* may well play a role in the photoperiod-refractory response. Encephalopsin (*OPN3*) was found to be highly expressed in the MBH (2.31 to 2.42 log2CPM) but without significant changes in expression. OPN3 has recently been identified in the hypothalamus of chick hatchlings [81] but not as yet to the MBH of adult birds. *OPN5* (− 0.46 to 0.89 log2CPM) and *VA* (− 0.11 to 0.31 log2CPM) were also unchanging and expressed at a low level in the MBH sample. These findings confirm the importance of temperature and photoperiod-dependent regulation of thyroid hormone metabolism in the avian MBH (Fig. 3).

### Quail immune gene repertoire

We investigated the immune genes in the quail genome in detail due to the importance of quail as a model in disease research. The MHC-B complex of the quail has been previously sequenced and found to be generally conserved compared to chicken in terms of gene content and order [30, 31]. However, the quail MHC contains a higher copy number of several gene families within the MHC-B [30] and shows increased structural flexibility [31], as well as an inversion in the *TAP* region [30]. The MHC-B sequence in the quail genome extends from the previously sequenced scaffold, and this additional region also contains similar gene content and order to chicken, but with gene copy number variations. As in the chicken, the *CD1A* and *B* genes are found downstream of the MHC I region, while many *TRIM* family genes and *IL4I1* are encoded upstream. The BG region, which encodes a family of butrophylin genes known as *BG* genes in the chicken, was also present in the quail. Within this region, six *BG* genes were identified in the quail, compared to 13 in the chicken [82]. At least five of these *BG* genes are transcribed in the quail lung and ileum. The chicken and turkey have an additional MHC locus known as the Rfp-Y or MHC-Y locus, which contains several copies of non-classical MHCI-Y and MHCIIB-Y genes. However, no MHC-Y genes have been previously identified in quail. BLAST searches of both the quail genome and quail transcriptomes, as well as the bobwhite and scaled quail genomes, failed to identify any MHC-Y genes, indicating this locus probably does not exist in the quail.

Cathelicidins and defensins are two families of antimicrobial peptides that have activities against a broad range of pathogens and exhibit immune-modulatory effects. Orthologs of all four chicken cathelicidins and of 13 chicken defensins [83] were identified in the quail genome (Additional file 11). Due to their high divergence, of the 13 defensins, only four were annotated through the annotation pipeline, with the remainder identified through BLAST and HMMer searches with chicken defensins. The only poultry defensin missing from the quail genome is *AvBD7*. The defensins are encoded in a 42 kb cluster on quail chromosome 3, as in chickens. A 4 kb gap in the scaffold in this region may explain the missing *AvBD7* sequence.

Several genes are thought to be crucial for influenza resistance in both humans and birds, including *RIG-I*, *TLR* and *IFITM* genes. *RIG-I* has not previously been identified in chicken, despite being present in ducks and many other bird orders, and is considered highly likely to be deleted from the chicken genome [84]. In addition, an important RIG-I binding protein RNF135 has also not been identified in chicken [85]. Likewise, an ortholog of *RIG-I* or *RNF135* could not be identified in the quail genome or transcriptomes through BLAST and HMMer searches and therefore is likely missing in the quail also. Orthologs of all five chicken *IFITM* genes (*IFITM1, 2, 3, 5* and *10*) were identified in the quail genome and transcriptomes. In addition, orthologs of each chicken toll-like receptors (TLRs), including key TLRs for viral recognition, *TLR4* and *TLR7*, were identified in the quail genome, except that of *TLR1A*. *TLR1A* was not identified through BLAST and HMMer searches of the quail genome. In chicken, *TLR1A* and *TLR1B* are located between the genes *KLF3* and *FAM11A1*. However, in the quail genome, there is only one gene at this location. We extracted TLR1-like sequences from other Galliform genomes and Zebrafinch and created a phylogeny with TLR2 and 4 as outgroups (Additional file 2: Figure S4). This phylogeny indicates single highly supported clades of TLR1A and B, indicating that the duplication occurred in an ancestor of Neognathae avians. TLR1A was identified in the other two quail species’ genomes. The absence of TLR1A from the quail genome assembly suggests it has been lost from the quail genome, although an assembly error cannot be ruled out.

### Quail response to H5N1 influenza

Highly pathogenic influenza A viruses (HPAI), such as strains of H5N1, are responsible for enormous economic losses in the poultry industry and pose a serious threat to public health. While quail can survive infection with low pathogenic influenza viruses (LPAI), they experience high mortality when infected with strains of HPAI [86]. Quail are more susceptible than chickens to infection by some strains of H5N1 including one that caused human mortality (A/Hong Kong/156/97) [36]. Previous research has shown that quail may play a key role as an intermediate host in the evolution of avian influenza, allowing viral strains to spread from wild birds to chickens and mammals [32, 33, 36, 87]. Unlike quail and chicken, aquatic reservoir species such as duck are tolerant of most HPAI strains [88]. The generation of a high-quality quail genome has enabled us to perform a differential transcriptomic analysis of gene expression in quail infected with LPAI and HPAI, to better understand the response of quail to influenza infection. Lung and ileum samples were collected at 1 day post infection (1dpi) and 3 days post infection (3dpi). We also reanalysed previous data collected from duck and chickens [89] and compare this to the quail response.

To provide an overview of the response to LPAI and HPAI in quail, we examined pathway and GO term enrichment of DEGs (see Additional file 12, Additional  file 13 and Additional file 2; Figures S5-S8). In response to LPAI infection, pathways enriched in the ileum included metabolism, JAK/STAT signalling, IL6 signalling and regulation of T cells (Additional file 2: Figure S5). In the lung, pathways upregulated included complement, IL8 signalling and leukocyte activation (Additional file 2: Figure S6). In the lung at 3dpi, highly enriched GO terms included “response to interferon-gamma”, “regulation of NF-kappaB”, “granulocyte chemotaxis” and “response to virus” (Additional file 2: Figure S7), which are key influenza responses. This indicates an active immune response occurs to LPAI infection in quail, involving both ileum and lung, but with the strongest immune response occurring in the lung.

Genes upregulated in response to HPAI in the ileum were related to metabolism and transport, while inflammatory response was downregulated at 1dpi (Additional file 2: Figure S7). Downregulated pathways at 1dpi included IL-6, IL-9 and neuro-inflammation signalling pathways (Additional file 2: Figure S7). In the quail lung, many genes were downregulated after HPAI infection (Additional file 12). At 3dpi, most downregulated pathways and terms were linked to immune system processes. GO terms with the highest fold enrichment in downregulated genes at this time included T and B cell proliferation, TNF signalling pathway, TLR pathway and IFN-G production (Additional file 13). Pathways downregulated included both Th1 and Th2 pathways, T cell, B cell and macrophage signalling pathways (Additional file 2: Figure S8). This indicates that crucial immune responses in quail are downregulated in ileum, and particularly in the lung at day 3, following HPAI infection.

To compare the response of quail, duck and chicken, clustering of gene counts was examined using BioLayout 3D [90]. This revealed a cluster of 189 genes that were strongly upregulated at 1dpi in the duck following HPAI infection, which showed no or very low response in chicken and quail (Additional file 14). This cluster was dominated by RIG-I pathway and IFN response genes including *IFNG*, *DDX60*, *DHX58*, *IRF1*, *IRF2* and *MX1*. Pathways associated with this cluster include MHCI processing and death receptor signalling (Additional file 2: Figure S9). Thus, the lack of this early anti-viral response may be key to the susceptibility of Galliformes to HPAI.

**Fig. 4:**
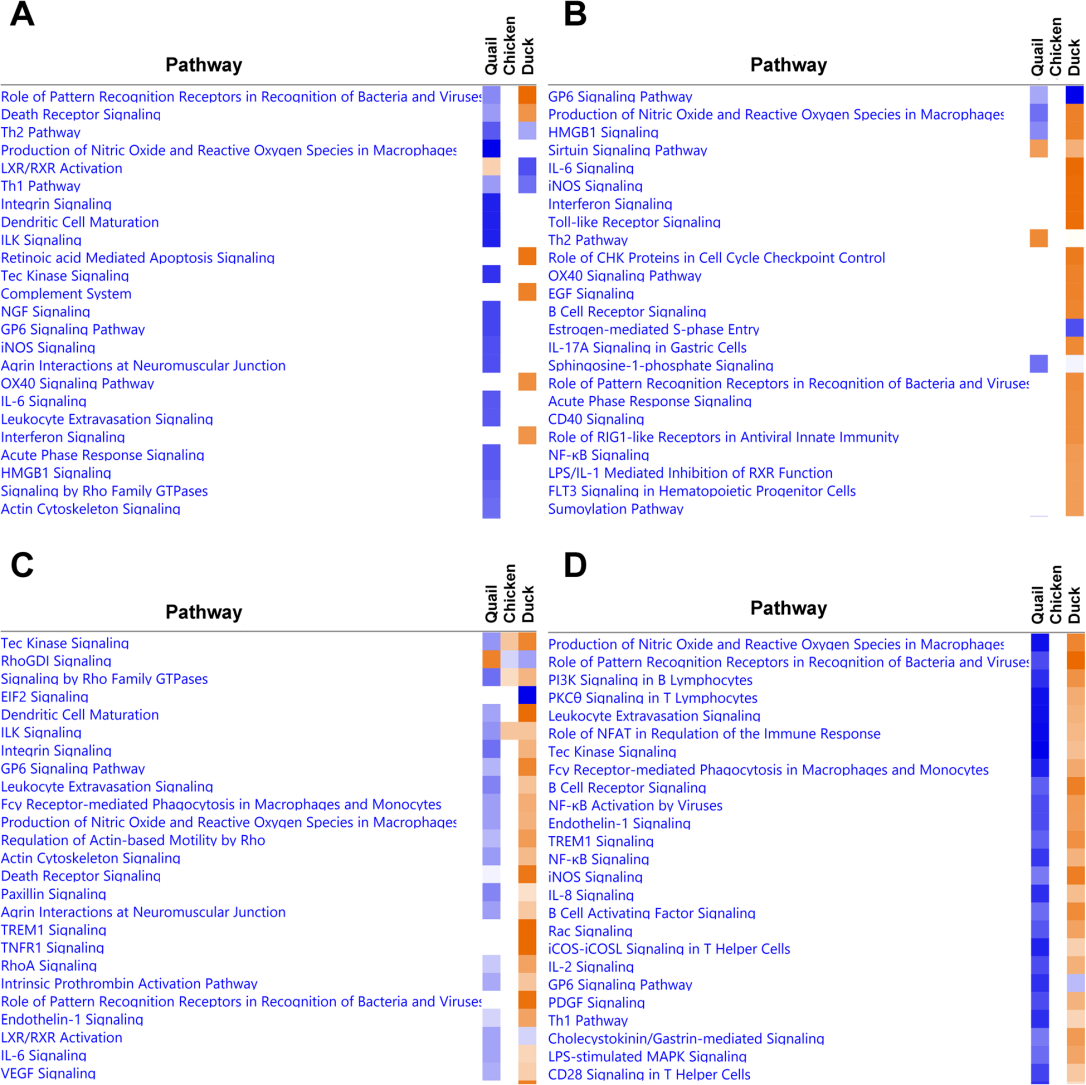
Heatmap comparison between pathways upregulated (orange) and downregulated (orange) in quail, chicken and duck following HPAI infection. Ileum day 1 **a**, ileum day 3 **b**, lung day 1 **c** and lung day 3 **d**

To further compare the responses between the three species, enrichment of pathways in each species was examined (Fig. 4; Additional file 2: Figure S10). In LPAI infection, comparison between ileum samples was limited due to low number of DEGs, but in lung, many pathways were shared between the species, primarily immune pathways. In HPAI, pathway analysis revealed very few commonly regulated pathways between the three species. However, at 1dpi in the ileum and 3dpi in the lung, there were many pathways that were downregulated in the quail, not altered in chicken and upregulated in the duck. In the ileum at 1dpi, this included pattern recognition and death receptor signalling. In the lung at 3dpi, this involved host of immune-related pathways including production of NOS by macrophages, pattern recognition, B and T cell signalling and NK-KB, IL8 and IL2 signalling.

**Fig. 5:**
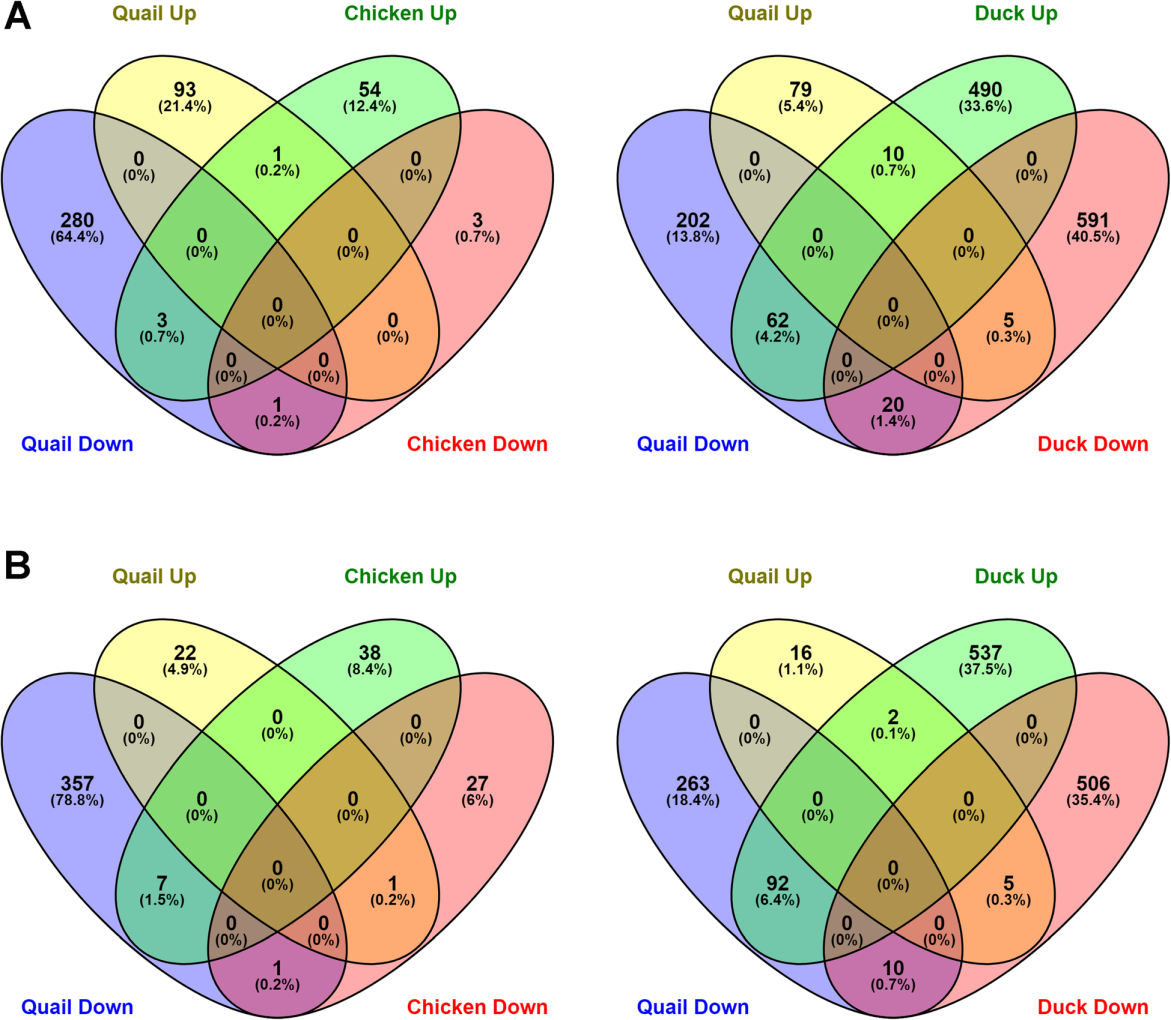
Proportion of genes commonly regulated between quail and chicken or duck to H5N1 infection on day 3. Ileum **a** and lung **b**

The proportion of genes commonly regulated between quail, chicken and duck to LPAI and HPAI infection was also examined (Fig. 5; Additional file 2: Figure S11). The responses to LPAI showed a high level of commonly regulated genes between the three species; for example, 50.5% of chicken DEGs and 42.5% of duck DEGs in lung at day 1 were also differentially expressed in quail. In HPAI, consistent with the heatmap comparison (Fig. 4), the responses of chicken, quail and duck were largely unique, with few genes commonly differentially expressed. There was a large set of genes that were upregulated in duck, while being downregulated in quail at 3dpi, in both ileum and lung. In lung, these genes were related primarily to innate immune system pathways, including pattern recognition pathways, cytokine production, leukocyte adhesion, TNF production, interferon production, B cell signalling and response to virus (Additional file 13). Genes with the greatest differential expression included *RSAD2* which inhibits viruses including influenza, *IFIT5* which senses viral RNA and *OASL* which has anti-viral activity. These differences further highlight that the anti-viral immune response is dysregulated in quail. Additionally in both ileum and lung, the apoptosis pathway was enriched in duck, but not in quail (Additional file 13). Apoptosis is known to be a critical difference in the response of chickens and ducks to HPAI infection [91].

Lastly, we examined the response of key families involved in influenza and immune response, focussing on the lung (Additional file 15). *IFITM* genes have previously been found to have a crucial role in HPAI resistance [89] and may block AIV from entering cells [92]. Consistent with previous findings in the chicken [89], quail showed no significant upregulation of *IFITM* genes, while these genes in duck were strongly upregulated (Additional file 15), TLRs and MHC receptors are involved in recognition of foreign molecules and triggering either an innate (TLR) or adaptive (MHC) immune response. TLR3, 4 and 7, which bind viral RNAs, were upregulated in response to LPAI in quail. A reversal was seen in response to HPAI, with *TLR4* and *7* substantially downregulated. Likewise, genes of both MHC class I and II were upregulated in response to LPAI and downregulated in response to HPAI. By comparison there was no perturbation of TLR and MHC genes in chicken and upregulation of class I genes in duck. The quail seems to have a highly dysfunctional response to HPAI infection with key innate and adaptive immune markers downregulated at 3dpi, which contrasts with the strong immune response mounted by the duck and minimal immune response in the chicken.

## Discussion

We have assembled, annotated and analysed a high-quality quail genome. Quails are a crucial model in developmental biology, behaviour and photoperiod research and also disease studies. Using this genome, we have made important discoveries in these fields of research.

The quail genome assembly is highly comparable to the chicken genome assembly (*Gallus gallus* 5.0) in terms of contiguity, assembly statistics, annotation, gene content and chromosomal organisation. It is also a superior assembly to other quail family and Galliform genome assemblies. The quail genome shows high conservation to the chicken both in chromosomal synteny, in gene orthology and in ERV genomic density. The immune gene complement in the quail genome is similar to that of chicken but with some important differences, including changes to the MHC including a likely lack of the MHC-Y locus and of the avian *TLR1A* gene.

Quail are used as a model to study the genetics of behaviour, and leveraging the quail genome we examined selection signatures in lines selected for sociability. This confirmed selection on regions harbouring genes known to be involved in human autistic disorders or related to social behaviour. Autistic spectrum disorders are observed in several disorders that have very different aetiology, including fragile X Syndrome, Rett Syndrome or Foetal Anticonvulsant Syndrome. While these disorders have very different underlying etiologies, they share common qualitative behavioural abnormalities in domains particularly relevant for social behaviours such as language, communication and social interaction [93, 94]. In line with this, several experiments conducted on high social (HSR) and low social (LSR) reinstatement behaviour quail indicate that the selection program carried out with these lines is not limited to selection on a single response, social reinstatement, but affect more generally the ability of the quail to process social information [18]. Differences in social motivation, but also individual recognition have been described between LSR and HSR quail [95, 96]. Inter-individual distances are longer in LSR quail [95] and LSR young quail have decreased interest in unfamiliar birds [97] and lower isolation distress than HSR ones [20]. Further experiments will be required to examine the possible functional link between the selected genes and the divergent phenotype observed in these lines. Also, by analyses of genes known to be differentially expressed in the zebra finch during song learning, we hope to comparatively understand molecular systems linked to behaviour in the avian brain.

Quail is a key model species for studying seasonal biology. We have added to this body of work by using the quail genome for genome-wide analysis to determine how photoperiod and temperature interact to determine the medial basal hypothalamus transcriptome. We confirm the importance of temperature and photoperiod-dependent regulation of thyroid hormone metabolism in the avian MBH. Temperature-dependent amplification and suppression of the photoperiod response may indicate qualitative differences in the MBH pathways or simply reflect different stages of progression through seasonally phased processes. This could be further investigated by contrasting across time-series at different temperatures. We also observed concurrent regulation of multiple hormonal signalling pathways, this may reflect a diversity of pathways and cell types in the MBH or reflect a corrective mechanism to account for cross-talk with other GPCR pathways. We observed LH, PRL, and GH receptor transcript changes which may indicate modulation of a GnRH-anterior pituitary feedback mechanism. In addition to observing high *OPN3* expression in the MBH, we also noted LD overexpression of *OPN4*, which could provide a potential component for an avian photoperiod-refractory mechanism. This study demonstrated the utility of genome-wide transcriptome analysis in quail to provide valuable insights and novel hypotheses for avian seasonal biology.

Quails are important for disease research, particularly in influenza where they act as a key intermediate host in the evolution of avian influenza [32–34], allowing viral strains to spread from wild birds to mammals and domesticated chickens. We found that quail have a robust immune response to infection with LPAI, allowing them to survive the infection. However, they show dysregulation of the immune response after infection with HPAI, and this may explain their susceptibility to HPAI strains. Quail, chicken and duck showed similar responses to LPAI. After HPAI infection, while ducks showed a robust immune response, quails did not. This difference may be a result of the higher viral dose the ducks were infected with; however, the lower dose given in chickens and quail still resulted in replicative virus and mortality of all chickens and quails by 5dpi, and therefore should have induced an anti-viral immune response. A more substantial immune response may have developed in the short period between 3dpi and time of death of the quails (between 3 and 4dpi); however, this was too late to prevent mortality. An IFITM response was not seen against HPAI while genes associated with apoptosis were downregulated, mechanisms previously found to be important in resistance to HPAI [89, 91], which potentially allows the virus to easily enter cells and spread early in infection. Anti-viral and innate immune genes, including those involved in antigen recognition, immune system activation and anti-viral responses were downregulated at 3dpi, which would prevent an effective immune response and viral clearance once infection is established. This study provides crucial data that can be used to understand the differing response of bird species to AIV, which will be critical for managing and mitigating these diseases in the future.

## Conclusions

Here we describe the assembly, annotation and use of a high-quality quail genome, an important avian model in biological and biomedical research. This genome will be crucial for future comparative avian genomic and evolutionary studies. It provides essential genetic and genomic reference information for making precise primers and nucleic acid probes, and accurate perturbation reagents including morpholinos, RNA inactivation tools, and CRISPR-Cas9 constructs. We have demonstrated the utility of this genome in both infectious disease and behavioural research providing further confirmation of the importance of quail as a research model, and for its role in agricultural and animal health studies. Specifically, the availability of this genome has allowed us to make significant discoveries in the unique response of quail to highly pathogenic avian influenza infection, helping elucidate the basis for extreme susceptibility seen in this species. It has also allowed us to identify and confirm genes and genomic regions associated with social behaviours. Furthermore, we have shown that genome-wide transcriptomics using this genome facilitated further insights and hypothesis into the mechanism of photoperiodism in avian seasonal biology. Moving forward, the availability of a high-quality quail genome will facilitate the study of diverse topics in both avian and human biology including disease, behaviour, comparative genomics, seasonality and developmental biology.

## Methods

### Whole genome sequencing and assembly

To facilitate genome assembly by avoiding polymorphism, we produced an individual as inbred as possible. We started with a quail line previously selected for early egg production and having a high inbreeding coefficient [98] and four generations of brother-sister matings produced a dedicated line “ConsDD” (*F* > 0.6) (PEAT, INRAE Tours, France). A 15-week-old male *Coturnix japonica* (id. 7356) was then selected from this line for the sequencing project. Genomic DNA was extracted from a blood sample using a high-salt extraction method [99]. Our sequencing plan followed the recommendations provided in the ALLPATHS2 assembler [37]. This model requires 45× sequence coverage of each fragment (overlapping paired reads ~ 180 bp length) from 3 kb paired-end (PE) reads as well as 5× coverage of 8 kb PE reads. These sequences were generated on the HiSeq2500 Illumina instrument. Long reads used for gap filling were generated at 20× coverage on the same DNA source using a RSII instrument (Pacific Biosciences). The Illumina sequence reads were assembled using ALLPATHS2 software [37] using default parameter settings and where possible, and scaffold gaps were closed by mapping and local assembly of long reads using PBJelly [100]. As most scaffold gaps were small, long-read data was only needed to correct around 1 Mb of the assembly. The Illumina long insert paired-end reads (3 kb and 8 kb PE) were used to further extend assembled scaffolds using SSPACE [101]. The draft assembly scaffolds were then aligned to the genetic linkage map [53] and the Galgal4.0 chicken reference (GenBank accession: GCA_000002315.2) to construct chromosome files following previously established methods [44]. Finally, all contaminating contigs identified by NCBI filters (alignments to non-avian species at the highest BLAST score obtained) and all contigs < 200 bp were removed prior to final assembly submission.

### Gene annotation

Specific RNA-Seq data for the genome annotation was produced from the same animal used for the genome assembly. RNA was extracted from heart, kidney, lung, brain, liver, intestine and muscle using Trizol and the Nucleospin® RNA II kit (MACHEREY-NAGEL), following the manufacturer’s protocol.

The *Coturnix japonica* assembly was annotated using the NCBI pipeline, including masking of repeats prior to ab initio gene predictions, for evidence-supported gene model building. We utilised an extensive variety of RNA-Seq data to further improve gene model accuracy by alignment to nascent gene models that are necessary to delineate boundaries of untranslated regions as well as to identify genes not found through interspecific similarity evidence from other species. A full description of the NCBI gene annotation pipeline was previously described [102]. Around 8000 lacked gene symbols from this pipeline, and these were further annotated manually by using BLAST searches using the corresponding sequences and extracting protein names from Uniprot.

### Comparative analyses

A set of single copy, orthologous, avian-specific genes were selected from OrthoDB v. 9 [42] and their status (present, duplicated, fragment or missing) were tested with BUSCO v.3.0.2 [43] in the *Gallus gallus* 5.0 and *Coturnix japonica* 2.0 genomes. Ab initio gene predictions were done within the BUSCO framework using tBLASTn matches followed by avian-specific gene predictions with Augustus v. 3.3 [103]. Gene status was assessed by running HMMER [104] with the BUSCO HMM profiles of the orthologous sequences. Comparative maps and breakpoint data were generated using AutoGRAPH [105] using chicken and quail gff annotation files, using default settings. The TLR1A phylogeny was constructed in MEGA7 [106] using the Neighbour-Joining method [107].

### Endogenous retrovirus identification

Endogenous retroviruses (ERVs) were identified in the *Coturnix japonica* 2.0 and Turkey 5.0 genome assemblies using the LocaTR identification pipeline [49] and compared to a previous analysis of ERVs in the *Gallus gallus* 5.0 genome assembly [44]. LocaTR is an iterative pipeline which incorporates LTR_STRUC [108], LTRharvest [109], MGEScan_LTR [110] and RepeatMasker [111] (http://repeatmasker.org) search algorithms.

### Sociability selection study

The data and methods used have been described previously [54]. Briefly, two quail lines were used, divergently selected on their sociability [19]: high social (HSR) and low social (LSR) reinstatement behaviour. A total of 10 individuals from generation 50 of each quail line were sequenced after equimolar DNA pooling. Sequencing was performed (paired-ends, 100 bp) on a HiSeq 2000 sequencer (Illumina), using one lane per line (TruSeq sbs kit version 3). The reads (190,159,084 and 230,805,732 reads, respectively, for the HSR and LSR lines) were mapped to the CoJa2.2 genome assembly using BWA [112], with the mem algorithm. Data are publicly available under SRA accession number SRP047364. Within each line, the frequency of the reference allele was estimated for all SNPs covered by at least 5 reads, using Pool-HMM [113]. This analysis provided 13,506,139 SNPs with allele frequency estimates in the two lines. FLK values [55] were computed for all these SNPs, and the local score method [54] was applied to the *p* value on single-marker tests.

### Photoperiod study

MBH tissue was collected as previously [75]. Male 4-week-old quail were obtained from a local dealer in Japan and kept under SD conditions (6L18D) for 4 weeks. At 8 weeks of age, quail were transferred to LD conditions (20L4D) and kept under LD conditions for 4 weeks to develop their testes. And then, 12-week-old LD quail were transferred to short-day and low-temperature (SL: 6L18D 9C) conditions for another 4 weeks to fully regress their testes. All samples were collected at 18 h after light on (ZT18), which for SD birds is 12 h after dark onset, and for LD birds 2 h before dark onset. (Lights on is same for LD and SD and lights off was extended in LD group). RNA-Seq was performed using a TruSeq stranded mRNA prep (Revision E 15031047) with 125 bp paired-end reads on a HiSeq Illumina 2500 with four replicates in each of the three conditions.

Reads were quality (Phred>25) and adapter trimmed with Trim Galore (version 0.4.5). Tophat (version 2.1.0) [114] with bowtie2 (version 2.2.6) was used to map reads to the quail genome (GCA_001577835.1 *Coturnix japonica* 2.0), using the NCBI annotation. We determined feature counts for gene loci using the featureCounts program [115] in the subread (version 1.5.0) package [116]. Statistical analysis was performed using the limma package [117] (version 3.36.1) in the R programming environment (version 3.5.0). The trimmed mean of M-values normalisation method (TMM) was used for normalisation with Voom for error estimation (Additional file 2: Figure S3). We retained gene loci with more than 10× coverage in three replicates in at least two conditions. A categorical least squared regression model was fitted using LD 23 °C, SD 23 °C and SD 9 °C conditions. Statistics for pairwise comparisons were then recalculated by refitting contrasts to the model for LD 23 °C vs SD 23 °C, LD 23 °C vs SD 9 °C and SD 23 °C vs SD. The Benjamini-Hochberg approach [118] was used to estimate the false discovery rate. For reporting numbers of photoperiod significant genes, we applied thresholds of FDR < 0.05, log2 CPM > 0 and absolute log2 fold change > 1. Temperature-dependent genes are reported as those with a photoperiod significant effect at either 23 °C or 9 °C and a significant effect when contrasting SD 9 °C and SD 23 °C at the same thresholds defined across photoperiods.

### Influenza response study

All experiments involving animals were approved by the Animal Care and Use Committee of St. Jude Children’s Research Hospital and performed in compliance with relevant policies of the National Institutes of Health and the Animal Welfare Act. All animal challenge experiments were performed in animal biosafety level 2 containment facilities for the LPAI challenges and in biosafety level 3 enhanced containment laboratories for the HPAI challenges. Viral challenges of quail, tissue collection, RNA extractions and sequencing were carried out as previously described for chicken [89]. Fifteen quail, 15 chickens and 15 ducks were challenged with 10^6^ EID_50_ intranasally, intratracheally and intraocularly of LPAI A/Mallard/British Columbia/500/2005 (H5N2) in phosphate buffered saline (PBS). Fifteen quail and 15 chickens were challenged with 10^1.5^ EID_50_ intranasally, intratracheally and intraocularly of HPAI A/Vietnam/1203/2004 (H5N1) in PBS. Twelve ducks were challenged with 10^6^ EID_50_ intranasally, intratracheally and intraocularly of HPAI A/Vietnam/1203/2004 (H5N1) in PBS. Mock infection control groups for quails (*n* = 12), chickens (*n* = 10) and ducks (*n* = 15) were also inoculated, receiving an equivalent volume of PBS via the same route of administration. Birds were randomly allocated to experimental groups. Oropharyngeal and cloacal swabs were taken from all birds and virus titres are shown in (Additional file 2: Tables S1–3). Animals were monitored daily for clinical signs. Lung and ileum samples were collected from all birds on 1dpi and 3 dpi. RNA extractions were performed using Trizol and QIAGEN’s RNeasy kit. For sequencing, 36-cycle single-ended sequencing was carried out on the Genome Analyser IIx using Illumina v3 Sequencing by Synthesis kits.

All quail, as well as duck and chicken RNA-Seq reads from the previous study [89], were analysed as follows: Ileum and lung RNAs were analysed from PBS infected control (3 samples from each of 1dpi and 3dpi), H5N1-infected (3 samples from each of 1dpi and 3dpi, except quail ileum 1dpi which had 2 samples) and H5N2-infected (3 samples from each of 1dpi and 3dpi). A total of 251 million reads of 36 nucleotides in length were generated for quail. Reads were quality checked using FastQC (version 0.11.2) and trimmed for quality using Trim Galore (version 0.4.0). Mapping was performed to the quail genome (GCA_001577835.1 Coturnix_japonica_2.0), chicken genome (GCA_000002315.3 Gallus_gallus-5.0) and duck (GCA_000355885.1 BGI_duck_1.0) using Tophat2 [114] (version 2.1.0) using default options including the default multi-mapping cutoff of 20 locations. Mapping of reads was also performed to H5N1 and H5N2 genomes using Kallisto [119] (version 0.42.4; Additional file 16). For quantification and differential analysis, the following pipeline was used. First, transcripts were assembled and quantified using cufflinks [120], guided with the NCBI annotation for the relevant genome, and the multi-read correct option was used to more accurately estimate abundances of multi-mapped reads. The transcriptomes were merged using stringtie merge [121], and cuffdiff [115] was used for differential analysis using default settings. To determine orthology between quail, duck and chicken genes, reciprocal BLAST searches were performed. For analysis of GO term enrichment, the PANTHER overrepresentation test [122] was used, and for pathway analysis, Ingenuity Pathway Analysis software (QIAGEN) was used. For clustering analysis, BioLayout 3D [90] was used using default settings except 1.4 inflation for Markov clustering.

## Supplementary information

Additional file 1. List of unannotated quail genes and their manual annotation

Additional file 2. Supplementary Figures. S1-S11 and Tables S1–3

Additional file 3. Location of breakpoints between chicken and quail chromosomes

Additional file 4. Percent of quail genes with orthologs identified in related bird genomes

Additional file 5. BED file containing location of ERVs in quail genome

Additional file 6. BED file containing location of ERVs in turkey genome

Additional file 7. A comparative summary of assembled ERVs in quail, chicken and turkey

Additional file 8. List of selection signatures detected from the HSR / LSR lines

Additional file 9. Statistics from photoperiod differential expression study

Additional file 10. Pathway analysis from photoperiod study

Additional file 11. Sequences of cathelicidins and defensin genes identified in quail genome

Additional file 12. List of differentially expressed genes, FDR < 0.05 and fold change > 1.6 in infection study

Additional file 13. Overrepresented GO terms in differentially expressed genes in infection study

Additional file 14. List of the cluster of genes upregulated in duck at day 1 during HPAI infection, with the corresponding fold changes in each species. NS = Not significantly differentially regulated

Additional file 15. Regulation of IFITM, MHC and TLR family genes in quail, chicken and duck following HPAI and LPAI infection

Additional file 16. Viral read presence in quail samples

## Data Availability

All data generated or analysed during this study are included in this published article (and its Additional files), or in the following public repositories. Data has been submitted to the public databses under the following accession numbers: genome sequence data, NCBI Genome [GCA_001577835.2] [123] (https://www.ncbi.nlm.nih.gov/assembly/GCA_001577835.2) and Ensembl [GCA_001577835.1] [124] (https://www.ensembl.org/Coturnix_japonica/Info/Index); transcription annotation data, SRA [PRJNA296888] [125] (https://www.ncbi.nlm.nih.gov//bioproject/PRJNA296888); RNA-seq data for infection studies, Array Express, quail [E-MTAB-3311] [126] (https://www.ebi.ac.uk/arrayexpress/experiments/E-MTAB-3311/), duck [E-MTAB-2909] [127] (https://www.ebi.ac.uk/arrayexpress/experiments/E-MTAB-2909/), chicken [E-MTAB-2908] [128] (https://www.ebi.ac.uk/arrayexpress/experiments/E-MTAB-2908/); Sequencing of HSR/LSR lines, SRA [SRP047364] [129] (https://www.ncbi.nlm.nih.gov/bioproject/PRJNA261665); RNA-seq data for photoperiod study, SRA [PRJNA490454] [130] (https://www.ncbi.nlm.nih.gov/bioproject/?term=PRJNA490454).
