# Supplementary material for: The quail genome: insights into social behaviour, seasonal biology and infectious disease response
Source: BMC Biol. 2020 Feb 12;18:14. doi: 10.1186/s12915-020-0743-4 (PMC7017630; doi:10.1186/s12915-020-0743-4)
Supplement: Supplementary file 2 — Supplementary Figures. S1-S11 and Tables S1–3 [file 12915_2020_743_MOESM2_ESM.docx]

Supplementary Figures


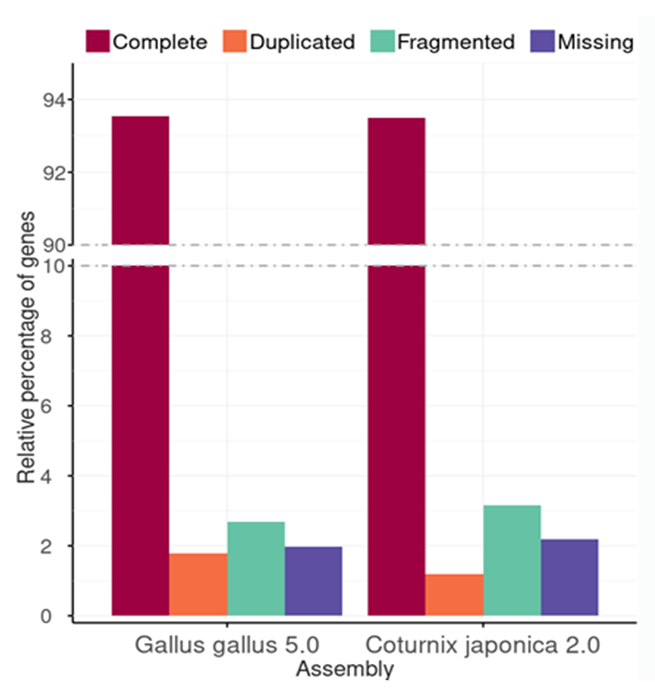


**Figure S1: Transcriptome and assembly completeness with BUSCO 3.**

Assessment of 4,915 single copy, orthologous, avian genes in two chicken and the quail genome assemblies were done using the BUSCO v. 3.0.2 pipeline. Complete genes are single copy orthologs; duplicated appear in multiple copies; fragmented are shorter than the expectation from the BUSCO profile match; missing is not found.


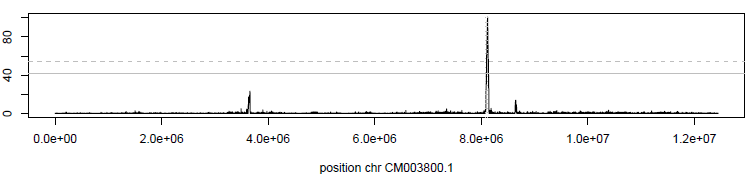


**Figure S2: Example of selection signature on quail chromosome 20.**

The local score (Lindley process based on the score function -log10(pFLK) - 1) is shown along genomic positions on chromosome 20 (bp).


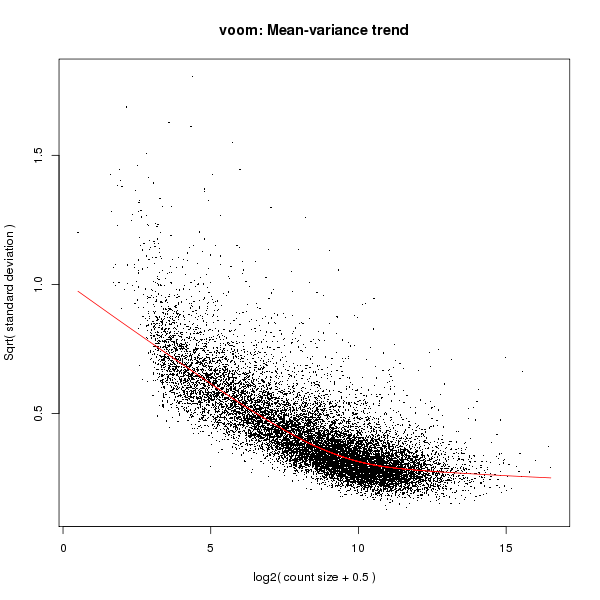


**Figure S3**: **Voom estimation of the mean-variance trend in the RNASeq**


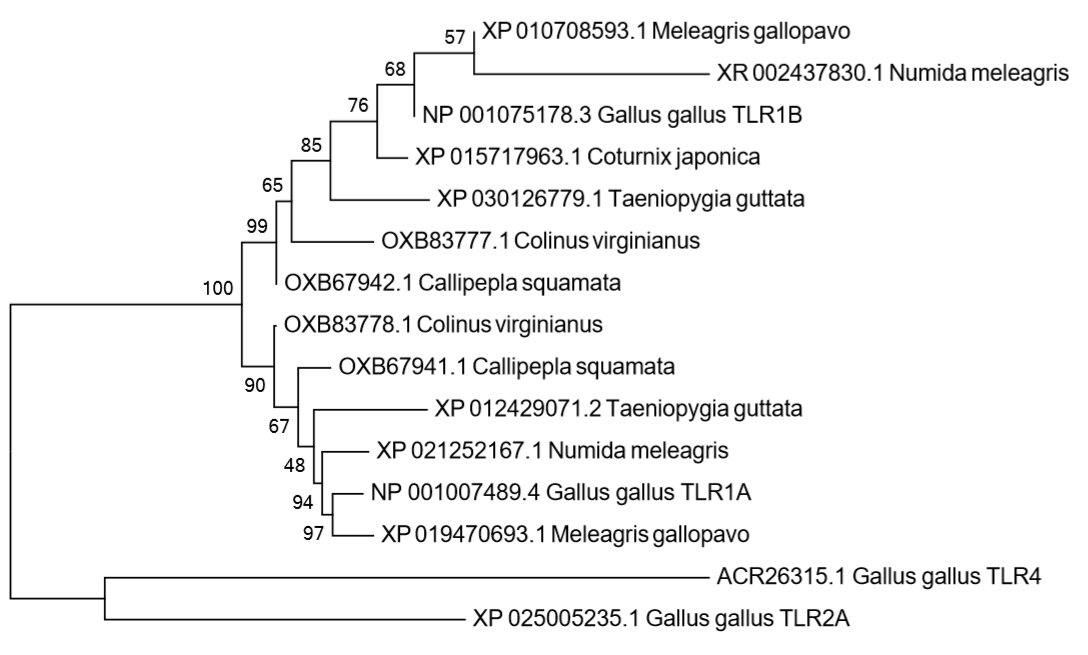


**Figure S4: Phylogenetic tree of TLR1A and B sequences in bird species**

The evolutionary history was inferred using the Neighbor-Joining method. The optimal tree with the sum of branch length = 2.81868810 is shown. The percentage of replicate trees in which the associated taxa clustered together in the bootstrap test (500 replicates) are shown next to the branches. All ambiguous positions were removed for each sequence pair. Chicken TLR2 and TLR$ used as outgroups. Species included: *Melagris gallopavo* (turkey), *Numida meleagris* (helmeted guinea fowl), *Gallus gallus* (chicken), *Coturnix japonica* (quail), *Taeniopygia guttata* (Zebrafinch), *Colinus virginianus* (Northern bobwhite) and *Callipepla squamata* (scaled quail).


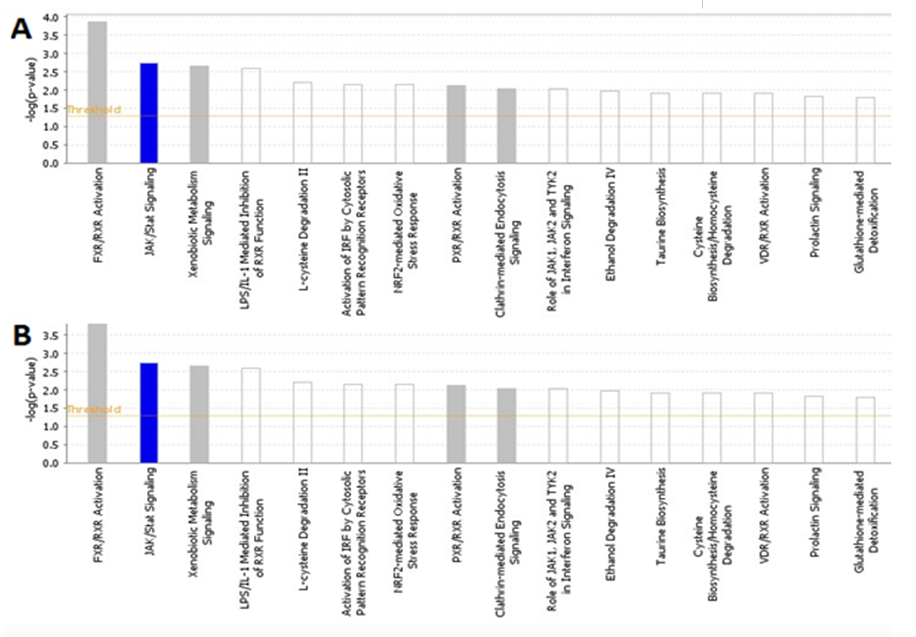


**Figure S5:**

Biological pathways significantly altered in the ileum of LPAI infected quail vs controls (1 day post infection (A) 3 days post infection (B))


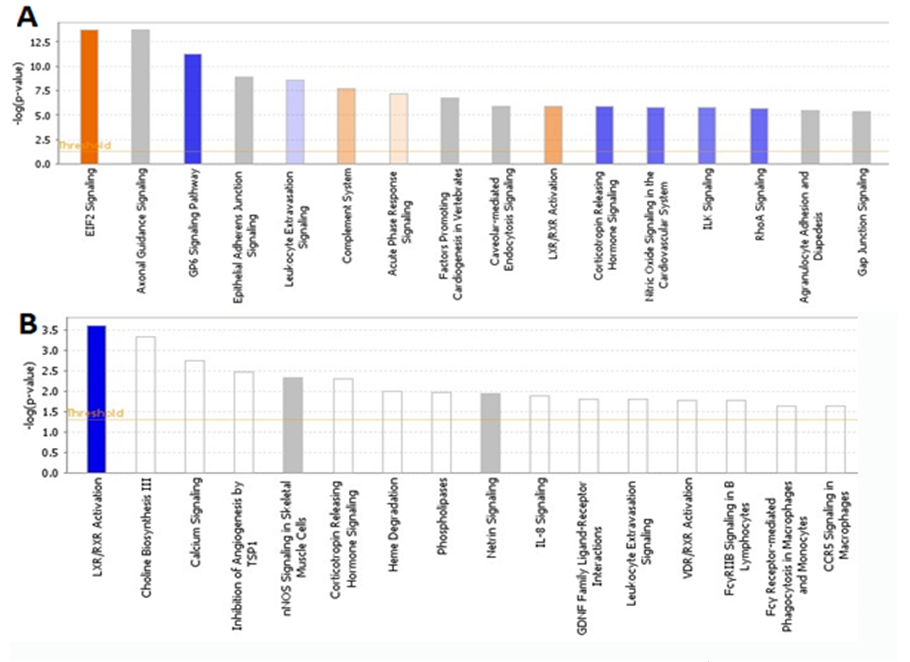


**Figure S6:**

Biological pathways significantly altered in the lung of LPAI infected quail vs controls (1 day post infection (A) 3 days post infection (B))


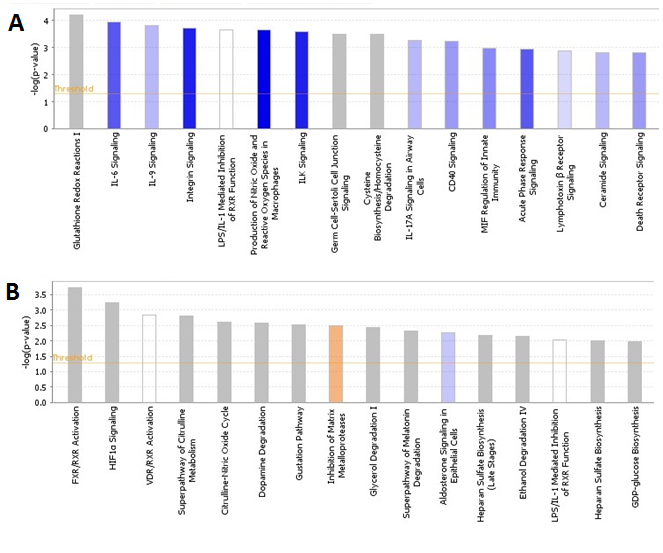


**Figure S7:**

Biological pathways significantly altered in the ileum of HPAI infected quail vs controls (1 day post infection (A) 3 days post infection (B))


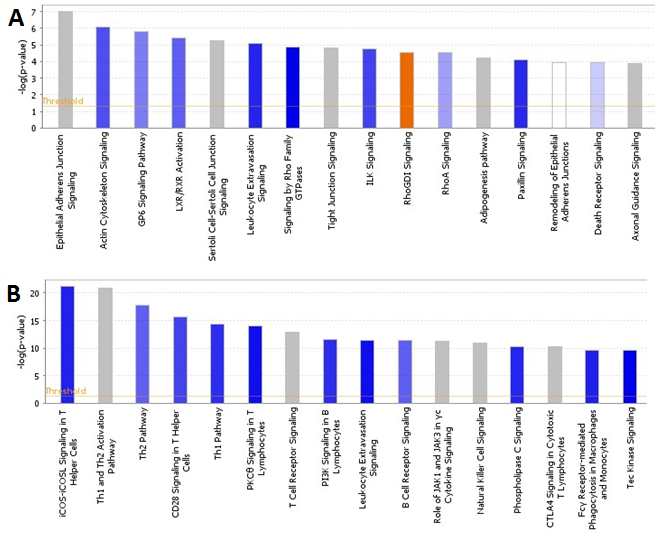
**Figure S8:**

Biological pathways significantly altered in the lung of HPAI infected quail vs controls (1 day post infection (A) 3 days post infection (B))


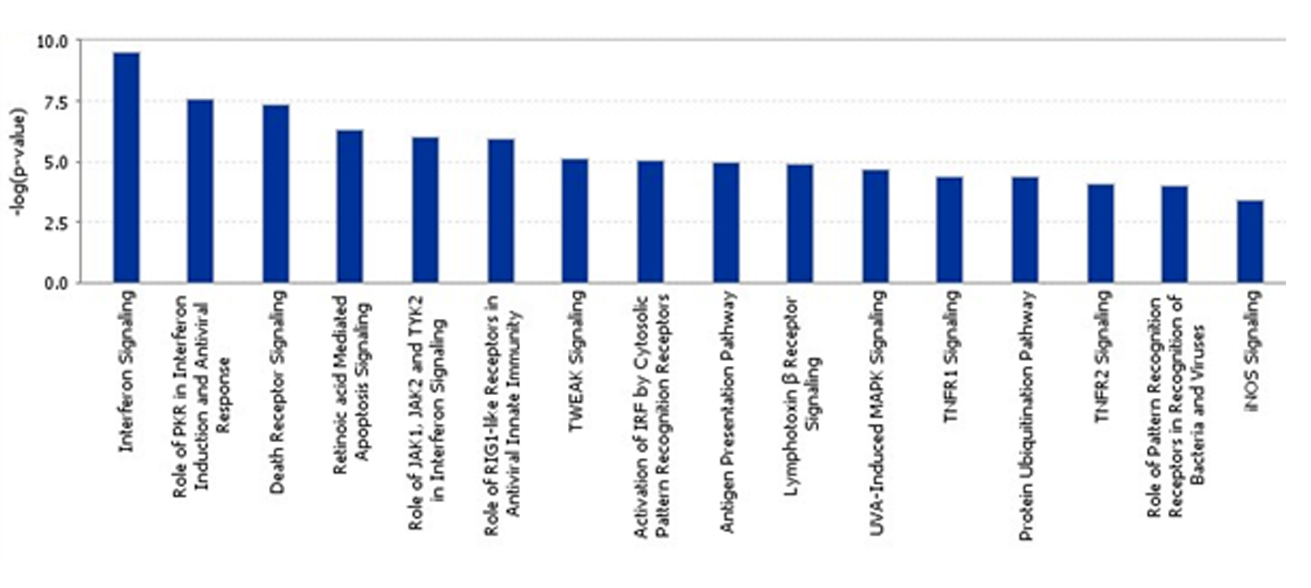


**Figure S9:** **Enriched pathways in a cluster of genes highly expressed in duck lung after HPAI infection**


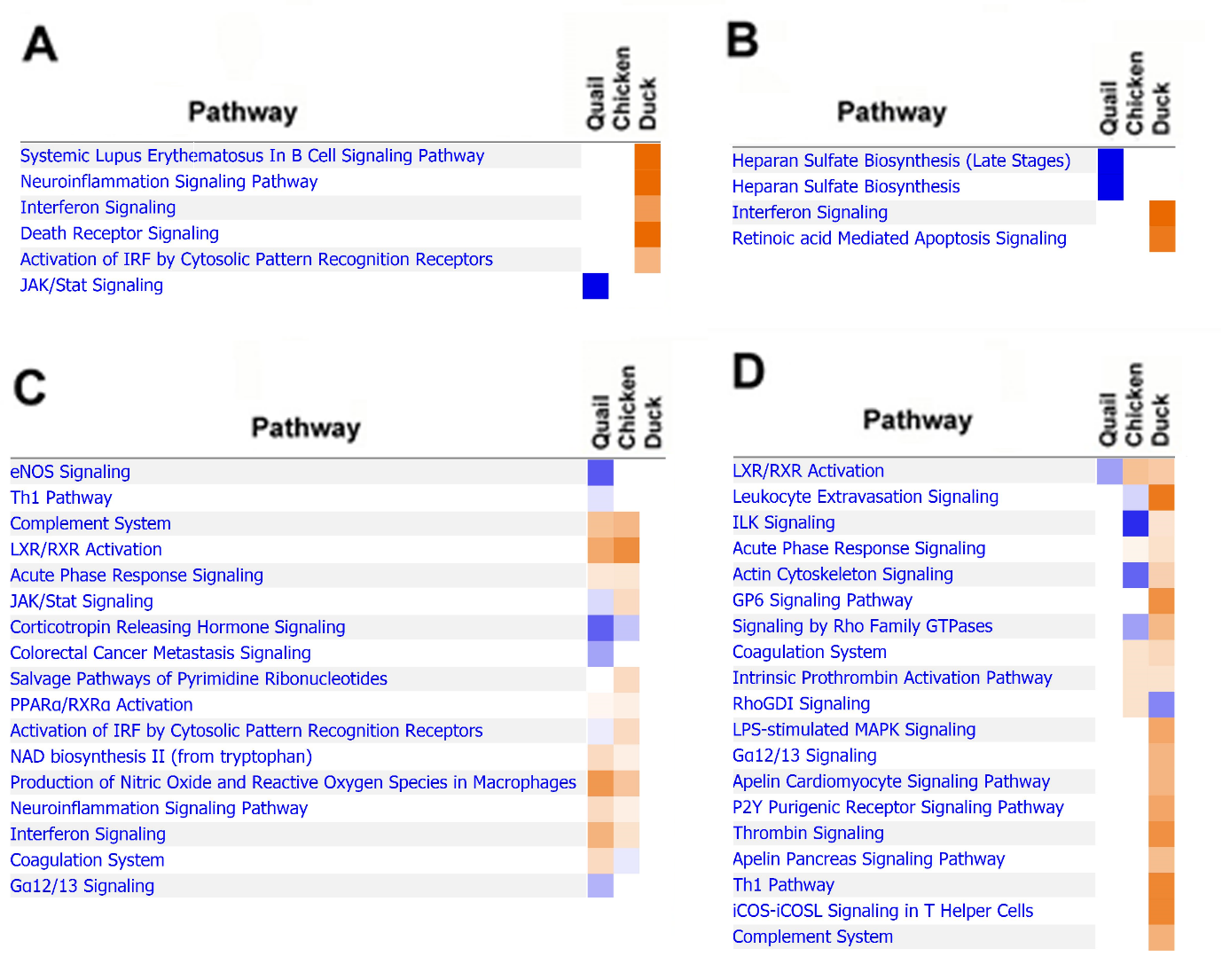


**Figure S10:** **Heatmap comparison between pathways upregulated (orange) and downregulated (orange) in quail, chicken and duck**

Ileum day 1 (A), ileum day 3 (B), lung day 1 (C) and lung day 3 (D).


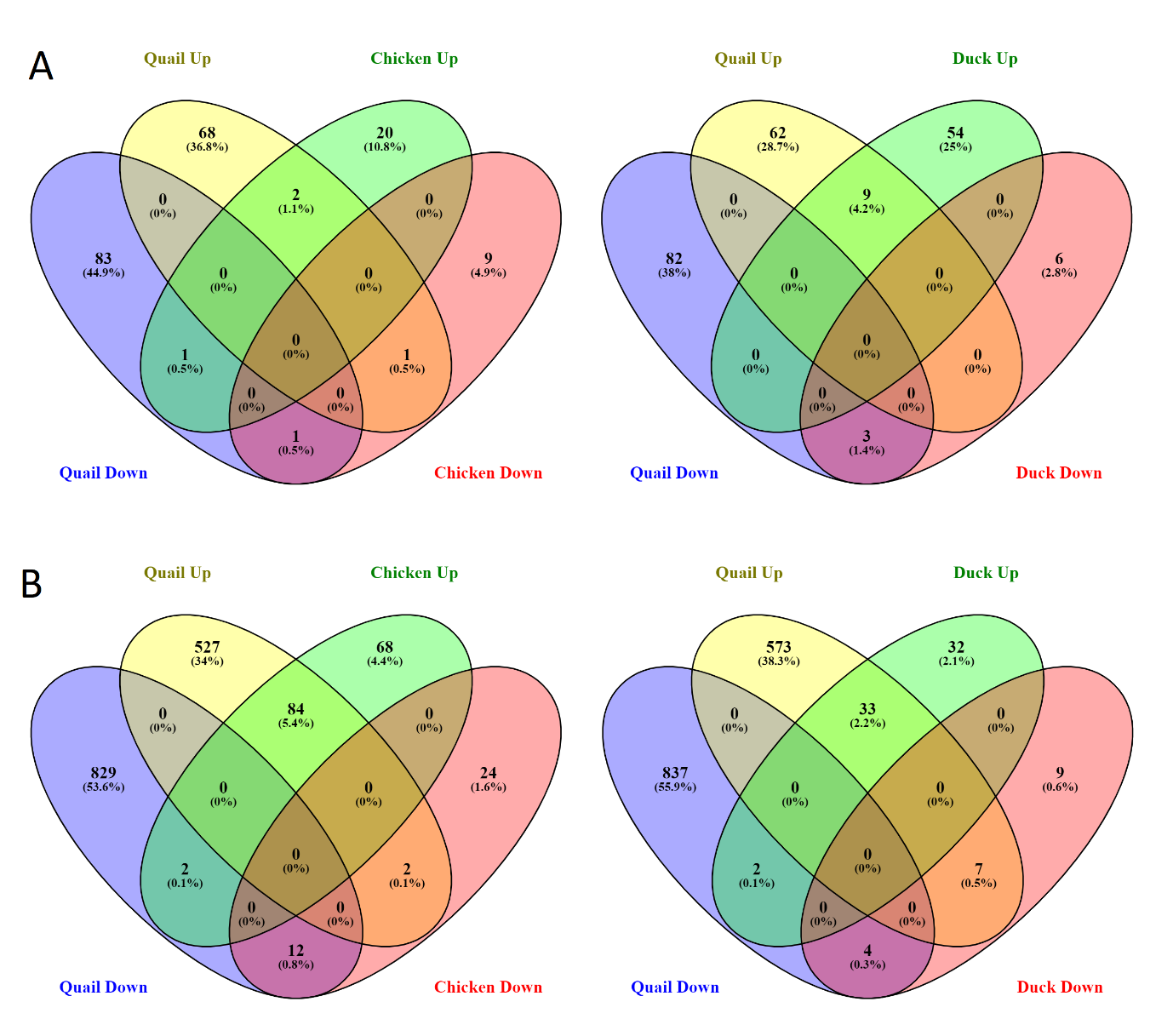


**Figure S11:** **Proportion of genes commonly regulated between quail and chicken or duck to LPAI infection on day 1**

Ileum (A) and lung (B)

**Table S1: Virus isolation in oropharyngeal and cloacal swabs from quail infected with A/Vietnam/1203/04 H5N1**

| **Quail ID #** | **Cloacal** | | | | **Oropharyngeal** | | | |
| --- | --- | --- | --- | --- | --- | --- | --- | --- |
|  | **2 DPI** | **5 DPI** | **7 DPI** | **9 DPI** | **2 DPI** | **5 DPI** | **7 DPI** | **9 DPI** |
| 2229^a^ |  |  |  |  |  |  |  |  |
| 2230^a^ |  |  |  |  |  |  |  |  |
| 2233^a^ |  |  |  |  |  |  |  |  |
| 2239^a^ |  |  |  |  |  |  |  |  |
| 2242^a^ |  |  |  |  |  |  |  |  |
| 2251^a, control^ |  |  |  |  |  |  |  |  |
| 2252^a, control^ |  |  |  |  |  |  |  |  |
| 2228^b^ | — |  |  |  | — |  |  |  |
| 2231^b^ | 2.7 |  |  |  | 4.7 |  |  |  |
| 2235^b^ | — |  |  |  | 5.5 |  |  |  |
| 2240^b^ | — |  |  |  | — |  |  |  |
| 2243^b^ | — |  |  |  | +/- |  |  |  |
| 2249^b, control^ | — |  |  |  | — |  |  |  |
| 2250 ^b, control^ | — |  |  |  | — |  |  |  |
| 2227^c^ | — |  |  |  | — |  |  |  |
| 2232^c^ | — |  |  |  | — |  |  |  |
| 2245^c^ | — |  |  |  | — |  |  |  |
| 2247^c, control^ | — |  |  |  | — |  |  |  |
| 2248^c, control^ | — |  |  |  | — |  |  |  |
| 2234 | 2 DPI | | | | | | | |
| 2236 | 2 DPI | | | | | | | |
| 2237 | — |  |  |  | 2.75 |  |  | 3 DPI |
| 2238 | — |  |  |  | — |  |  | 4 DPI |
| 2241 | — |  |  |  | 2.25 |  |  | 4 DPI |
| 2244 | — |  |  |  | — |  |  | 4 DPI |
| 2246 | 2.25 |  | | | 2.5 | 3 DPI | | |

Oropharyngeal and cloacal swabs from all birds were negative for virus isolation 0 days post infection (DPI)

The lower limit of detection of virus in the upper respiratory tract was 0.75 log_10_EID_50_/ml. Results are shown as individual virus titers of swabs in log_10_EID_50_/ml.

+/- result were positive in 1-3 out of 3 eggs inoculated at undilute but less that the limit of detection when titrated.

^control^Quail inoculated with phosphate buffered saline (PBS) only

^a^Sacrificed for organs 1 DPI

^b^Sacrificed for organs 2 DPI

^c^Sacrificed for organs 3 DPI

Quail found dead or euthanized prior to conclusion of experiment. The day post infection that animal was found dead or euthanized is indicated in white text.

**Table S2: Virus isolation in oropharyngeal and cloacal swabs from chicken infected with A/Vietnam/1203/04 H5N1**

| **Chicken ID #** | **Cloacal** | | | | **Oropharyngeal** | | | |
| --- | --- | --- | --- | --- | --- | --- | --- | --- |
|  | **2 DPI** | **5 DPI** | **7 DPI** | **9 DPI** | **2 DPI** | **5 DPI** | **7 DPI** | **9 DPI** |
| 101^a^ |  |  |  |  |  |  |  |  |
| 103^a^ |  |  |  |  |  |  |  |  |
| 108^a^ |  |  |  |  |  |  |  |  |
| 109^a^ |  |  |  |  |  |  |  |  |
| 110^a^ |  |  |  |  |  |  |  |  |
| 124^a, control^ |  |  |  |  |  |  |  |  |
| 125^a, control^ |  |  |  |  |  |  |  |  |
| 102^b^ | 4.75 |  |  |  | 5.5 |  |  |  |
| 104^b^ | — |  |  |  | — |  |  |  |
| 105^b^ | — |  |  |  | — |  |  |  |
| 106^b^ | — |  |  |  | — |  |  |  |
| 107^b^ | — |  |  |  | — |  |  |  |
| 123^b, control^ | — |  |  |  | — |  |  |  |
| 112^c^ | — |  |  |  | — |  |  |  |
| 115^c^ | — |  |  |  | — |  |  |  |
| 117^c^ | — |  |  |  | — |  |  |  |
| 119^c^ | +/- |  |  |  | — |  |  |  |
| 120^c^ | — |  |  |  | — |  |  |  |
| 121^c, control^ | — |  |  |  | — |  |  |  |
| 122^c, control^ | — |  |  |  | — |  |  |  |
| 111 | — |  |  |  | — |  |  | 3 DPI |
| 113 |  |  |  |  |  |  |  | 2 DPI |
| 114 | — |  |  |  | — |  |  | 5 DPI |
| 116 | — |  |  |  | — |  |  | 3 DPI |
| 118 | 3.5 |  |  |  | 4.64 |  |  | 3 DPI |

Oropharyngeal and cloacal swabs from all birds were negative for virus isolation 0 days post infection (DPI)

The lower limit of detection of virus in the upper respiratory tract was 0.75 log_10_EID_50_/ml. Results are shown as individual virus titers of swabs in log_10_EID_50_/ml.

+/- result were positive in 1-3 out of 3 eggs inoculated at undilute but less that the limit of detection when titrated.

^control^Chicken inoculated with phosphate buffered saline (PBS) only

^a^Sacrificed for organs 1 DPI

^b^Sacrificed for organs 2 DPI

^c^Sacrificed for organs 3 DPI

Chicken found dead or euthanized prior to conclusion of experiment. The day post infection that animal was found dead or euthanized is indicated in white text.

**Table S3: Virus isolation in oropharyngeal and cloacal swabs from duck infected with A/Vietnam/1203/04 H5N1**

| **Duck ID #** | **Cloacal** | | | | **Oropharyngeal** | | | |
| --- | --- | --- | --- | --- | --- | --- | --- | --- |
|  | **2 DPI** | **5 DPI** | **7 DPI** | **9 DPI** | **2 DPI** | **5 DPI** | **7 DPI** | **9 DPI** |
| 51^a^ |  |  |  |  |  |  |  |  |
| 52^a^ |  |  |  |  |  |  |  |  |
| 53^a^ |  |  |  |  |  |  |  |  |
| 54^a^ |  |  |  |  |  |  |  |  |
| 55^a^ |  |  |  |  |  |  |  |  |
| 71^a, control^ |  |  |  |  |  |  |  |  |
| 72^a, control^ |  |  |  |  |  |  |  |  |
| 73^a, control^ |  |  |  |  |  |  |  |  |
| 74^a, control^ |  |  |  |  |  |  |  |  |
| 57^b^ | 2.75 |  |  |  | 5.5 |  |  |  |
| 59^b^ | 2.5 |  |  |  | 4.25 |  |  |  |
| 62^b^ | +/- |  |  |  | 3.83 |  |  |  |
| 63^b^ | 3 |  |  |  | 4.5 |  |  |  |
| 66^b^ | 3.5 |  |  |  | 5.5 |  |  |  |
| 75^b, control^ | — |  |  |  | — |  |  |  |
| 76 ^b, control^ | — |  |  |  | — |  |  |  |
| 77 ^b, control^ | — |  |  |  | — |  |  |  |
| 78 ^b, control^ | — |  |  |  | — |  |  |  |
| 56 | +/- | — | — | — | 5.5 | 2.75 | — | — |
| 58 | +/- | 2.25 |  |  | 3.75 | 2.5 |  | 6 DPI |
| 60 | 2.5 | — | — | — | 3.5 | — | — | — |
| 61 | +/- | — |  |  | 5.25 | 3.5 |  | 6 DPI |
| 64 | 1.75 | — |  |  | 3.25 | +/- |  | 6 DPI |
| 65 | +/- | — | — | — | 3.5 | +/- | — | — |
| 67 | 1.75 |  |  |  | 3.75 |  |  | 5 DPI |
| 68 | 3.75 |  |  |  | 5.25 |  |  | 4 DPI |
| 69 | 1.67 |  |  |  | 5.75 |  |  | 4 DPI |
| 70 | 2.5 | +/- | — | — | 5.75 | 1.75 | — | — |

Oropharyngeal and cloacal swabs from all birds were negative for virus isolation 0 days post infection (DPI)

The lower limit of detection of virus in the upper respiratory tract was 0.75 log_10_EID_50_/ml. Results are shown as individual virus titers of swabs in log_10_EID_50_/ml.

+/- result were positive in 1-3 out of 3 eggs inoculated at undilute but less that the limit of detection when titrated.

^control^Ducks inoculated with phosphate buffered saline (PBS) only

^b^Sacrificed for organs 3 DPI

Ducks found dead or euthanized prior to conclusion of experiment
